# The structural mechanism of HIV-2 Vif antagonism of human APOBEC3H

**DOI:** 10.1038/s41467-026-75045-1

**Published:** 2026-06-29

**Authors:** Yange Niu, Michelle Lilly, Estelle K. Ronayne, Michael Emerman, Nicholas M. Chesarino, John D. Gross

**Affiliations:** 1 Department of Pharmaceutical Chemistry, University of California San Francisco, San Francisco, CA USA; 2 Divisions of Human Biology and Basic Sciences, Fred Hutchinson Cancer Center, Seattle, WA USA; 3 Biophysics Graduate Program, University of California San Francisco, San Francisco, CA USA; 4 Department of Biology, University of Akron, Akron, OH USA; 5 Quantitative Bioscience Institute, University of California San Francisco, San Francisco, CA USA

**Keywords:** Cryoelectron microscopy, RNA-binding proteins, Virus-host interactions, Viral immune evasion, Restriction factors

## Abstract

Human APOBEC3 (A3) proteins restrict retrovirus infection by inducing hypermutations in viral cDNA. To counteract this restriction, lentiviruses, such as HIV-1 and HIV-2 encode the viral infectivity factor (Vif), which hijacks a host Cullin-RING E3 ubiquitin ligase complex to target A3 proteins for proteasomal degradation. Here, we present the cryo-EM structure of HIV-2 Vif in complex with human A3H and CBFβ. The structure reveals that A3H forms a dimer mediated by dsRNA where each A3H monomer directly interacts with an HIV-2 Vif and the host protein CBFβ. Both HIV-2 Vif-A3H and CBFβ-A3H interfaces are critical for A3H degradation. Notably, however, the HIV-2 Vif-A3H interface is entirely distinct from the previously determined cryo-EM structure of the HIV-1 Vif and A3H complex. These findings suggest that HIV-1 and HIV-2 Vif, which are the result of distinct cross-species transmissions from species with different A3H characteristics, have followed separate evolutionary trajectories to counteract human A3H.

## Abstract (web summary)

HIV-2 Vif counteracts a broad range of APOBEC3 restriction factors to promote viral infectivity. Here, the authors determine the structure of HIV-2 Vif bound to human APOBEC3H and the host cofactor CBFβ, uncovering a distinct mode of APOBEC3H recognition and dimerization.

## Introduction

The APOBEC3 (A3) family of cytidine deaminases constitutes a critical intrinsic antiviral defense in the human innate immune system^1–4^. A3 proteins restrict retroviral replication by incorporating into budding virions through interactions with viral RNA, then inducing G to A hypermutations during reverse transcription, thereby inhibiting productive infection as well as through direct inhibition of reverse transcription^5–11^. Among the seven human A3 members, A3D, A3F, A3G, and A3H are potent inhibitors of retroviruses, including HIV-1 and HIV-2^12,13^. A3D, A3F, and A3G contain two cytidine deaminase (CDA) domains, whereas A3H possesses a single CDA domain and represents the most evolutionarily divergent A3 protein^12,14,15^.

Human A3H exists in seven haplotypes with distinct protein stabilities and antiviral activities^14,16^. Three haplotypes, such as A3H hapII, encode stable, cytoplasmically localized proteins and strongly inhibit retroviral replication. The other four haplotypes, such as A3H hapI, are unstable, encode proteins that are rapidly degraded and have weak antiviral activity^17,18^. The stable A3H variants are more frequent in African populations but uncommon in other human populations^14,19^. Recent structures of human A3H hap II, chimpanzee A3H, and pig-tailed macaque A3H reveal that A3H forms a unique dimer mediated by double-stranded RNA (dsRNA)^20–23^ that is essential for A3H cytoplasmic localization and incorporation into virions^20^. Because of their potent restriction capacity and high polymorphism, stable A3H variants represent a major barrier to viral adaptation from non-human primates to humans^19,24,25^.

To overcome A3-mediated restriction, primate lentiviruses such as HIV-1, HIV-2, and SIV express the accessory protein viral infectivity factor (Vif), which hijacks the host transcription cofactor CBFβ and the Cullin-RING E3 ubiquitin ligase complex to polyubiquitinate A3 proteins for proteasomal degradation^26–32^. Vifs isolated from people living with HIV-1 (PLWH) that are homozygous for unstable A3H haplotypes are inefficient at degrading stable A3H haplotypes, such as hapII. Vif proteins isolated from PLWH who are homozygous for stable A3H alleles exhibit increased activity against active A3H, highlighting the ongoing evolutionary arms race between host and virus^19,25,31,33^. In contrast to HIV-1, HIV-2 Vif isolates tested to date exhibit broad antagonistic activity, efficiently targeting both A3G and stable A3H haplotypes^25,34,35^.

Diversifying selection of A3 and viral adaptation of Vif proteins play a critical role in determining viral host range and cross-species transmission. HIV-1 evolved from SIV infecting chimpanzees (SIVcpz), which itself arose from recombination between two different SIV strains: one from red-capped mangabeys (SIVrcm) and another from mustached monkeys (SIVmus). However, the *Vif* gene was contributed from the SIVrcm region and required adaptive changes in Vif to overcome A3-mediated restriction during transmission from Old World monkeys to chimpanzees, and then to humans^36–41^. In contrast, HIV-2 originated from SIV infecting sooty mangabeys (SIVsmm), whose Vif appears pre-adapted to antagonize human A3G and A3H, enabling transmission without additional Vif evolution^35,37,40,42,43^. While the structural mechanisms of HIV-1 Vif antagonism of A3G, A3H, and A3F_CTD_ have been elucidated, the structural basis of HIV-2 Vif antagonism towards human A3 proteins remains unknown^44–49^.

Here, we report the cryo-EM structure of HIV-2 Vif in complex with human A3H hap II and CBFβ, which reveals the interactions between A3H, Vif, and CBFβ. Structural and biochemical analyses uncover distinct binding modes of A3H with HIV-1 and HIV-2 Vif, as well as differences in dimer organization between the two complexes, providing detailed molecular insights into how HIV-2 Vif evolved towards antagonism of human A3H through different interfaces than HIV-1.

## Results

### Architecture of A3H-Vif-CBFβ complex

We screened a series of HIV-2 Vif sequences from both major subtypes of HIV-2 for their ability to express in insect cells and selected the HIV-2 ALI primary isolate strain for further biochemical characterization (Supplementary Fig. 1a). Wild-type HIV-2 ALI Vif exhibits robust inhibition of A3H packaging into virus like particles (VLPs) and maintains high infectivity in the presence of A3H, comparable to the well described lab-adapted HIV-2 strain (ROD) and the A3H-adapted HIV-1 strain (1203), but stronger than the HIV-1 strain (LAI) (Supplementary Fig. 1b, c)^25^. The Vif-E3 ligase is a modular protein complex that is comprised of a substrate receptor (Vif, CBFβ, Elongin B (ELOB), and Elongin C (ELOC) or VCBC) and a Cullin backbone (Cullin-5/Rbx2). To facilitate structural studies, wild-type human A3H hapII was co-expressed with VCBC and the N-terminal domain of Cullin-5 (residues 1-386, CUL5N), using a baculovirus expression system. The complex was expressed in insect cells through baculovirus infection and purified for cryo-EM analysis (Supplementary Fig.1d, e).

**Fig. 1:**
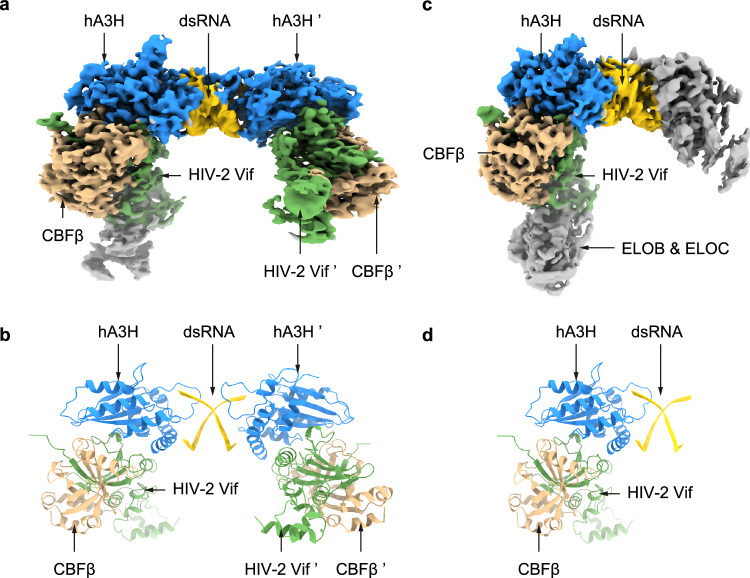
Cryo-EM structure of A3H-Vif-CBFβ complex. Cryo-EM density map **a** and atomic model **b** of the dimeric A3H-Vif-CBFβ complex. Cryo-EM density map **c** and atomic model **d** of the monomeric A3H-Vif-CBFβ complex. HIV-2 Vif is shown in green, hA3H in blue, CBFβ in light brown, and dsRNA in yellow. Unmodeled density is depicted in grey.

Data collection and processing yielded a 3.2 Å resolution reconstruction, enabling us to build an atomic model of the complex (Fig. 1, Supplementary Figs. 2–3 and Supplementary Table 1). The densities of A3H, HIV-2 Vif, and CBFβ (collectively referred to as A3H-Vif-CBFβ) were sufficient to build a reliable model. In contrast, no density was observed for CUL5N, and the densities corresponding to ELOB and ELOC were too weak for reliable model building, likely reflecting their intrinsic flexibility (Fig. 1a, c). The A3H-Vif-CBFβ complex forms a dimer bridged by a double-stranded RNA (dsRNA), which copurified with the complex and was protected from RNase digestion used during the protein purification. Each A3H monomer binds to one Vif-CBFβ subcomplex, and no direct interactions are observed between the two Vif-CBFβ units (Fig. 1a, b). A3H dimerization is mediated solely by dsRNA, and the overall dimer arrangement of A3H closely resembles the previously reported A3H crystal structure, with a root mean square deviation (RMSD) of 2.84 Å (Supplementary Fig. 4)^22^. The dsRNA is a 7-bp duplex with an additional nucleotide overhang, consistent with prior A3H-RNA structures^21–23^. Notably, unlike the HIV-1 Vif-A3G complex, RNA does not participate directly in the interface between A3H, HIV-2 Vif, and CBFβ (Fig. 1).

Among the two A3H-Vif-CBFβ copies, one is better resolved than the other, likely due to relative motion within the dimer. We therefore applied focused refinement using a mask around one monomer, which improved the local resolution (Supplementary Figs. 2 and 3). The resulting densities for A3H-Vif-CBFβ and dsRNA are well defined, enabling accurate model building and detailed analysis of their interactions.

### Interactions required for viral infectivity line the interface between A3H and HIV-2 Vif

**Fig. 2:**
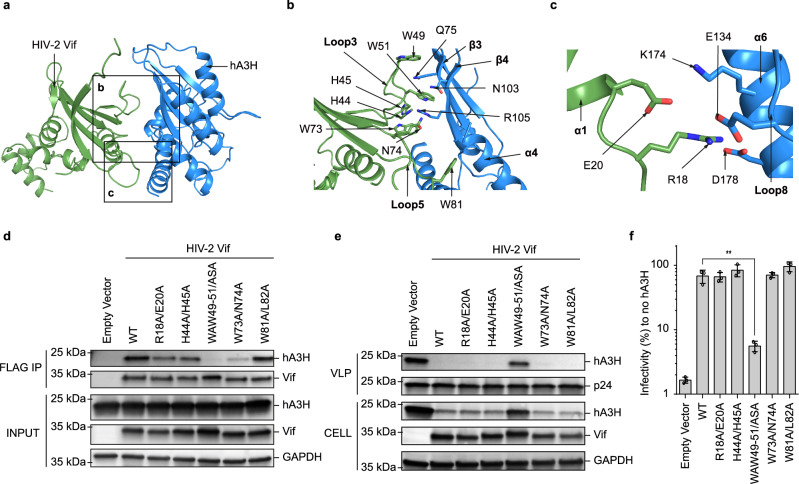
HIV-2 Vif-hA3H binding interface and functional characterization. **a** Structure of the HIV-2 Vif-hA3H subcomplex. HIV-2 Vif and hA3H are colored as in Fig.1. **b**, **c** Close-up views of the HIV-2 Vif-hA3H interface. The residues involved in interaction are shown as sticks. **d** Co-immunoprecipitation analysis of hA3H binding to HIV-2 Vif mutants. **e** Immunoblot analysis of hA3H incorporation into virus-like particles (VLPs) in the presence of HIV-2 Vif mutants. **f** Effects of HIV-2 Vif mutants on HIV-1 ΔVif infectivity in the presence of hA3H. The Y-axis is the % of the relative light units (RLUs) of the viral infections from virus generated in the presence of A3H relative to viral infections from virus generated in the absence of A3H. The viruses were normalized to equal reverse transcriptase activity prior to infection. Data are shown as means ± standard deviations; *n* = 3 biologically independent samples. Significance was determined using unpaired two-tailed Student’s *t*-tests (**p = 0.0024). **d**, **e** were repeated twice independently with similar results. Source data are provided as Source data file.

The interaction between A3H and Vif-CBFβ is primarily mediated by contacts between A3H and HIV-2 Vif, with a small interface between A3H and CBFβ further strengthening the complex. The interface between A3H and HIV-2 Vif has a buried solvent accessible surface area of 1055.11 Å², consistent with a tight interaction. This interaction is primarily mediated by loop3, loop5, and α1 of HIV-2 Vif, and by β3-β4 sheets and α4-α6 helices of A3H (Fig. 2a–c). At the core of the interface, several aromatic and polar residues establish key contacts (Fig. 2b). On Vif loop3, the indole ring of W49 forms a polar-π interaction with the amide group of Q75 on A3H β3 (Fig. 2b). Nearby, W51 of Vif engages in dual interactions: a polar-π interaction with the amide group of N103 and a cation-π interaction with the guanidinium group of R105, both located on A3H β4 (Fig. 2b). R105 of A3H additionally forms hydrogen bonds with the imidazole nitrogen of H44 on Vif, the amide group of N74 on Vif loop5, and participates in a cation-π interaction with the indole ring of W73 on Vif loop5 (Fig. 2b). Additional contacts occur between loop5 of Vif and α4 of A3H, where the W81 side chain interacts with the main chain of G128 (Fig. 2b). Electrostatic interactions may further stabilize the interface: the guanidinium group of R18 on Vif α1 forms salt bridges with the carboxylate groups of E134 on A3H loop8 and D178 on A3H α6, while the carboxylate group of E20 on Vif α1 interacts with the terminal amino group of K174 on A3H α6 (Fig. 2c).

To assess the functional relevance of these interactions, we introduced mutations in HIV-2 Vif and evaluated their effects on A3H binding (Fig. 2d), A3H packaging (Fig. 2e) and infectivity assays (Fig. 2f). Consistent with the structural observations, the R18A/E20A and W73A/N74A double mutations of Vif partially reduced interactions with A3H, whereas the WAW49-51/ASA mutations nearly abolished binding (Fig. 2d). Previous studies have also highlighted the importance of the first three residues of tryptophan-rich motif (WXWW, X any amino acid, Trp-rich motif) on Vif loop3 for HIV-2 Vif antagonism specifically against A3H^34^. In agreement with these findings, the WAW49-51/ASA mutation in Vif impaired its ability to promote degradation of A3H in cells, increased A3H packaging, and reduced viral infectivity (Fig. 2e, f). Notably, this Trp-rich motif is conserved in SIVsmm Vif, the precursor of HIV-2 Vif (Supplementary Fig. 5a). Introducing the same WAW49-51/ASA mutations into SIVsmm Vif similarly weakened its interaction with A3H, reduced Vif-mediated degradation in cells and increased packaging of A3H into VLPs, mirroring the effects observed for HIV-2 Vif (Supplementary Fig. 5b, c). Moreover, although the R18A/E20A and W73A/N74A Vif double mutants exhibit reduced interaction with A3H, the remaining interaction is sufficient to drive A3H degradation and does not measurably affect A3H packaging or viral infectivity (Fig. 2e, f). Collectively, these results demonstrate that WAW49-51 residues are critical for HIV-2 and SIVsmm Vif antagonism of A3H.

### HIV-2 Vif orients CBFβ for interaction with A3H

**Fig. 3:**
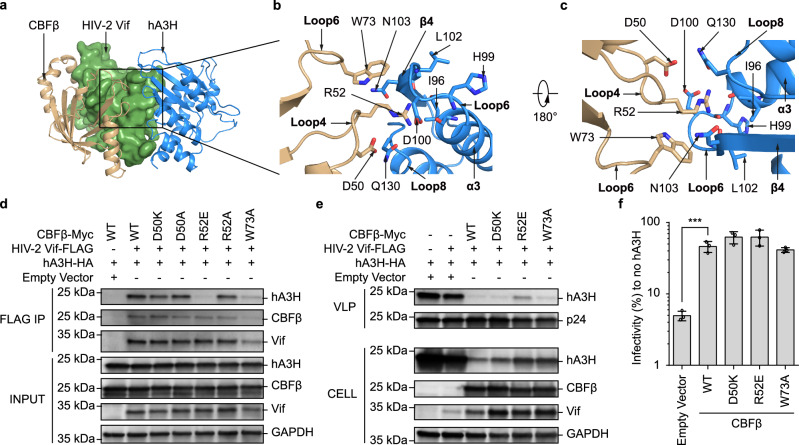
CBFβ-hA3H binding interface and functional characterization. **a** Structure of the HIV-2 Vif-CBFβ-hA3H complex. Subunits are colored as in Fig. 1. HIV-2 Vif is shown in surface representation, with the first seven N-terminal residues omitted for clarity. **b** Close-up view of the CBFβ-hA3H interface with interacting residues shown as sticks. **c** A 180° rotated view of (**b**). **d** Co-immunoprecipitation of CBFβ mutants and hA3H in the presence of HIV-2 Vif. **e** Immunoblot analysis of hA3H incorporation into VLPs in the presence of HIV-2 Vif and CBFβ mutants. **f** Effect of CBFβ mutants on HIV-1 ΔVif infectivity in the presence of HIV-2 Vif and hA3H. Viruses were produced in 293T cells knocked down for endogenous CBFβ and then complemented with empty vector, CBFβ WT, or CBFβ mutants. The *Y*-axis is the % of the RLUs of the viral infections from virus generated in the presence of A3H relative to viral infections from virus generated in the absence of A3H. The viruses were normalized to equal reverse transcriptase activity prior to infection. Data are shown as means ± standard deviations; *n* = 3 biologically independent samples. Significance was determined using unpaired two-tailed Student’s *t*-tests (***p = 0.0009). **d**, **e** were repeated twice independently with similar results. Source data are provided as Source data file.

We also identified an interface mediating the interaction between A3H and CBFβ, with a buried solvent-accessible surface area of 299.67 Å² (Fig. 3a–c). This interface involves loop4 and loop6 of CBFβ and loop6, β4, and loop8 of A3H (Fig. 3b, c). At this interface, the carboxylate group of D50 on CBFβ loop4 forms a hydrogen bond with the amide group of Q130 on A3H loop8 (Fig. 3b, c). The side chain of R52 on CBFβ loop4 inserts into a negatively charged pocket of A3H, where its guanidinium group forms an electrostatic interaction with the carboxylate group of D100 on A3H loop6, and hydrogen bonds with the main chain carbonyl oxygens of I96 on A3H α3, H99 on A3H loop6 and L102 on A3H β4 (Fig. 3b, c and Supplementary Fig. 6a). Additionally, the indole ring of W73 on CBFβ loop6 engages in a polar-π interaction with the amide group of N103 on A3H β4 (Fig. 3b, c). Notably, these interactions are unique to the HIV-2 Vif-A3H complex and are absent in the HIV-1 Vif-A3H and HIV-1 Vif-A3G structures (Supplementary Fig. 6b, c)^44–46,48^.

To validate the A3H-CBFβ interactions identified in our HIV-2 structure, we evaluated the effects of CBFβ mutants transfected into cells knocked down for endogenous CBFβ, the HEK293T-shCBFβ stable cell line described previously (Supplementary Fig.6d)^30^. Viral infectivity is abolished in this knockdown cell line in the presence of HIV-1 or HIV-2 Vif, indicating that the CBFβ knockdown is sufficient to impair Vif function (Fig. 3f and Supplementary Fig. 6g). Mutations in CBFβ diminish its interaction with A3H, with the R52E substitution exhibiting the most pronounced effect (Fig. 3d). The CBFβ R52E mutation reduces HIV-2 Vif dependent A3H degradation and modestly restores A3H incorporation into VLPs, whereas it has minimal effects on HIV-1 Vif dependent A3H degradation and packaging (Fig. 3e and Supplementary Fig. 6f). This is consistent with structural observations and biochemical results showing that R52 of CBFβ does not directly contact A3H in the HIV-1 Vif complex (Supplementary Fig. 6b, e)^48,49^. Nonetheless, viral infectivity is largely unchanged for viruses produced in the presence of the CBFβ R52E mutation with either HIV-1 or HIV-2 Vif, indicating that this interaction is not essential for HIV-2 Vif mediated antagonism of A3H (Fig. 3f and Supplementary Fig. 6g). However, it may nonetheless contribute to the affinity or specificity of the HIV-2 Vif-A3H complex.

### The binding modes of A3H with HIV-1 and HIV-2 Vif are distinct

**Fig. 4:**
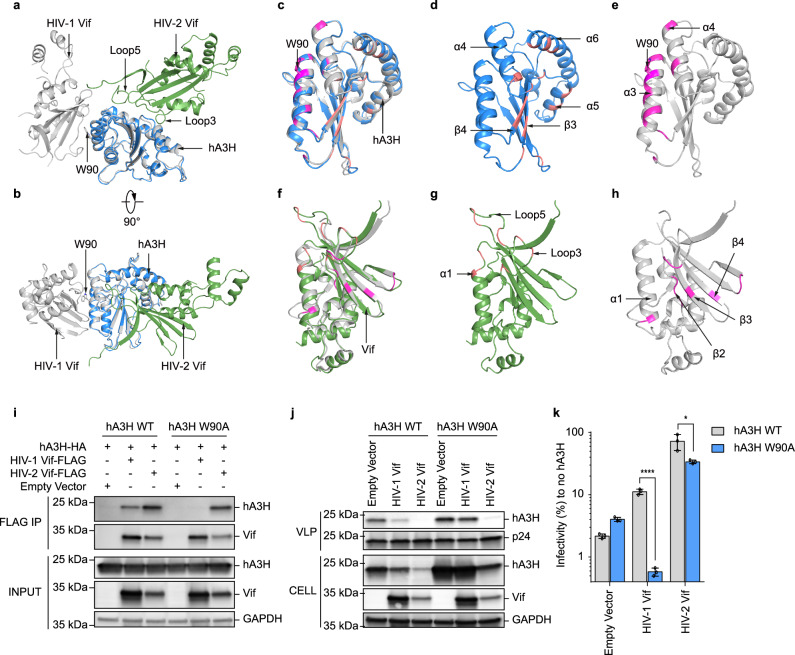
Structural comparison of HIV-2 and HIV-1 Vif in complex with hA3H. **a** Structural superposition of the HIV-2 Vif-hA3H complex (colored) with the HIV-1 Vif-hA3H complex (grey; PDB: 8FVI) aligned by hA3H. The hA3H W90 residue is shown as sticks. **b** A 90° rotated view of (**a**). **c** Superposition of hA3H in the HIV-2 Vif (green) and HIV-1 Vif (grey) complexes. hA3H residues contacting HIV-2 and HIV-1 Vif are colored as salmon and magenta, respectively. **d** HIV-2 Vif binding interface on hA3H, comprising β3-β4 and helices α4-α6. **e** HIV-1 Vif binding interface on hA3H, comprising helices α3 and α4. **f** Structural alignment of HIV-2 Vif (green) and HIV-1 Vif (grey). HIV-2 Vif residues contacting hA3H are highlighted in salmon; HIV-1 Vif interface residues are in magenta. **g** hA3H-binding interface on HIV-2 Vif, comprising loop 3, loop 5, and helix α1. **h** hA3H-binding interface on HIV-1 Vif, comprising β2-β4 and helix α1. **i** Co-immunoprecipitation of HIV-2 and HIV-1 (LAI) Vif with hA3H WT and W90A mutant. **j** Immunoblot analysis of hA3H WT and W90A incorporation into VLPs in the presence of HIV-2 or HIV-1 (LAI) Vif. **k** Effect of the hA3H W90A mutation on HIV-1 ΔVif infectivity in the presence of HIV-2 or HIV-1 (LAI) Vif. The *Y*-axis is the % of the RLUs of the viral infections from virus generated in the presence of A3H WT or A3H W90A relative to viral infections from virus generated in the absence of A3H. The viruses were normalized to equal reverse transcriptase activity prior to infection. The HIV-1 Vif is from the LAI strain, while the HIV-2 Vif is from the ALI strain. Data are shown as means ± standard deviations; *n* = 3 biologically independent samples. Significance was determined using unpaired two-tailed Student’s *t*-tests (*p = 0.0332, ****p < 0.0001). **i**, **j** were repeated twice independently with similar results. Source data are provided as Source data file.

We superimposed our HIV-2 Vif-A3H structure with the previously determined HIV-1 Vif-A3H complex, and found that HIV-2 and HIV-1 Vif engage A3H through completely different interaction interfaces (Fig. 4a, b, Supplementary Fig. 7a, b, and Supplementary Movie 1)^48,49^. Structural alignment using A3H as the reference revealed that the overall A3H fold is conserved between the two complexes, with an RMSD of 0.59 Å, indicating that A3H does not undergo significant conformational changes upon binding to either Vif (Fig. 4c). In the HIV-2 complex, the interaction surface on A3H is extensive, involving the β3-β4 sheets and α4-α6 helices (Fig. 4d). By contrast, in the HIV-1 complex, the interface is much smaller and is mainly confined to the α3-α4 region of A3H (Fig. 4e). Structural alignment using Vif as the reference further highlighted these differences (Fig. 4f). In HIV-2 Vif, the A3H-binding surface is largely composed of loop regions, particularly loop3 and loop5 (Fig. 4g). In contrast, the interface in HIV-1 Vif is primarily formed by β-sheets, including β2-β4 (Fig. 4h). Although the overall folds of HIV-1 and HIV-2 Vif are similar (RMSD = 1.2 Å), notable conformational differences are observed in loop5 and helix α1 (Supplementary Fig. 7c, d). In HIV-2 Vif, loop5 shifts outward toward A3H, enabling additional contacts; for example, T76 (corresponding to T74 of HIV-1 Vif) moves 7.8 Å outward to establish tight interactions with A3H (Supplementary Fig. 7c). Moreover, the N-terminal portion of helix α1 in HIV-2 Vif partially unwinds into a loop and shifts toward A3H, forming electrostatic contacts (Supplementary Fig. 7d). In particular, R18 (corresponding to R17 of HIV-1 Vif) moves 8.6 Å outward toward A3H (Supplementary Fig. 7d). These conformational rearrangements in HIV-2 Vif position the protein in an optimal configuration to form extensive and tight interactions with A3H.

Previous structural studies of the HIV-1 Vif-A3H complex identified W90 of A3H as a critical residue for interaction with HIV-1 Vif^48,49^. Mutation of W90 renders A3H fully resistant to HIV-1 Vif-mediated antagonism^48,49^. In our HIV-2 Vif-A3H structure, W90 is positioned away from the Vif interface and does not directly contact HIV-2 Vif (Fig. 4a, b). To examine the functional relevance of this difference, we generated the A3H W90A mutant and tested the ability of HIV-1 and HIV-2 to antagonize its activity. As reported, W90A disrupts interaction with HIV-1 Vif, resulting in resistance to HIV-1 Vif-dependent degradation (Fig. 4i, j). In contrast, W90A maintains strong interaction with HIV-2 Vif and remains sensitive to HIV-2 Vif-mediated degradation and exclusion from viral-like particles (VLPs) (Fig. 4i, j). Consistently, viral infectivity in the presence of HIV-1 Vif is fully restricted by A3H containing the W90A mutation, whereas in the presence of HIV-2 Vif it is not (Fig. 4k). Moreover, unlike the HIV-2 Vif complex with CBFβ and A3H, there are minimal interactions between CBFβ and A3H when bound to HIV-1 Vif (Supplementary Fig. 6b, c). These results confirm that A3H interacts with HIV-1 and HIV-2 Vif through distinct interfaces.

### Conserved A3H interactions between HIV-2 and SIVsmm Vif

As the precursor of HIV-2, SIVsmm can cross the species barrier from sooty mangabey monkeys to humans without requiring adaptation. A3H sequences are highly conserved across Old World monkeys and primate species, and the residues involved in interaction with HIV-2 Vif are identical between hA3H and smmA3H, except for a single D178E substitution (Supplementary Fig. 8a). Structural modeling indicates that this substitution does not alter the interaction with HIV-2 Vif, indicating that A3H evolution is unlikely to have been driven by selective pressure from HIV-2 Vif. Similarly, the Vif sequences of HIV-2 and SIVsmm are highly conserved, and the residues that contact A3H are identical, demonstrating that the Vif-A3H interface is maintained between HIV-2 and its precursor virus (Supplementary Fig. 8b). Residues in HIV-2 Vif that are essential for hA3H binding and antagonism are also crucial for SIVsmm Vif function (Supplementary Fig. 5a, b). In contrast, many of the HIV-2 Vif residues at the A3H interface are not conserved in HIV-1 Vif and SIV Vif derived from hominid primates (SIVcpz and SIVgor), suggesting that HIV-1 and hominid SIV Vifs do not exploit the same interface used by HIV-2 Vif to antagonize A3H (Supplementary Fig. 8b). Interestingly, SIV Vif proteins from other Old World monkeys, such as rhesus macaques (mac), African green monkeys (agm), and red-capped mangabeys (rcm), contain a Trp-rich motif and share key interface residues critical for A3H interaction, indicating that the HIV-2 Vif-A3H interface observed in our structure may be evolutionarily conserved among Old World monkeys (Supplementary Fig. 8b). The marked differences in interface residues and binding modes between HIV-2 and HIV-1 Vif in complex with A3H are consistent with these viruses following distinct evolutionary trajectories.

### Ubiquitination of A3H by HIV-2 Vif

Substrate ubiquitination involves the formation of an isopeptide bond between the C-terminal carboxyl group of glycine (G76) on ubiquitin and the amino group of lysine residues on the substrate^50^. To identify potential ubiquitination sites on A3H, we superimposed the Cullin-RING E3 ligase and ubiquitin-loaded E2 complex onto our HIV-2 Vif-A3H structure (Supplementary Fig. 9a, b)^51–53^. The C-terminal G76 residues of ubiquitin are positioned in close proximity to both A3H monomers, suggesting that each A3H subunit could be ubiquitinated simultaneously and subsequently targeted for proteasomal degradation (Supplementary Fig. 9a, b). Several lysine residues on the A3H surface are within reach of the G76 residue of ubiquitin, including K27, the K-rich motif (K50-K52), K64, and K153 (Supplementary Fig. 9a, b). Among these, K50 and K51 have been previously identified as major ubiquitination sites mediating HIV-1 Vif-dependent degradation of both chimpanzee and human A3H^48,49^. In addition, K27 and K153 have been confirmed as ubiquitinated residues by mass spectrometry^49^. These findings indicate that the ubiquitination sites targeted by HIV-1 and HIV-2 Vif are largely conserved. Interestingly, K64 lies closest to the G76 residue of ubiquitin in our model but has not been reported as a ubiquitination site in HIV-1 Vif-mediated degradation by mass spectrometry (Supplementary Fig. 9a, b)^49^. To investigate this, we performed Vif-mediated A3H degradation assays using lysine to arginine mutants to assess the contribution of individual lysine residues. Mutations within the K-rich motif substantially impaired HIV-2 Vif-mediated A3H degradation, indicating that this region serves as the primary ubiquitination site for HIV-2 Vif and represents a conserved ubiquitination site required for both HIV-1 and HIV-2 Vif-mediated A3H degradation (Supplementary Fig. 9c). The K27R and K64R mutants exhibited partial resistance to HIV-2 Vif-mediated degradation, suggesting that K27 and K64 also contribute as additional ubiquitination sites (Supplementary Fig. 9c). This observation suggests that HIV-2 Vif may engage a broader set of ubiquitination sites on A3H than HIV-1 Vif, potentially contributing to its stronger antagonistic activity. Notably, K64 is conserved among human and Old World monkey A3H, consistent with the broad antagonism exerted by HIV-2 Vif across species (Supplementary Fig. 8a). Collectively, these data indicate that HIV-2 Vif utilizes conserved ubiquitination sites shared with HIV-1 Vif, while also potentially expanding the range of modified residues to enhance A3H degradation.

**Fig. 5:**
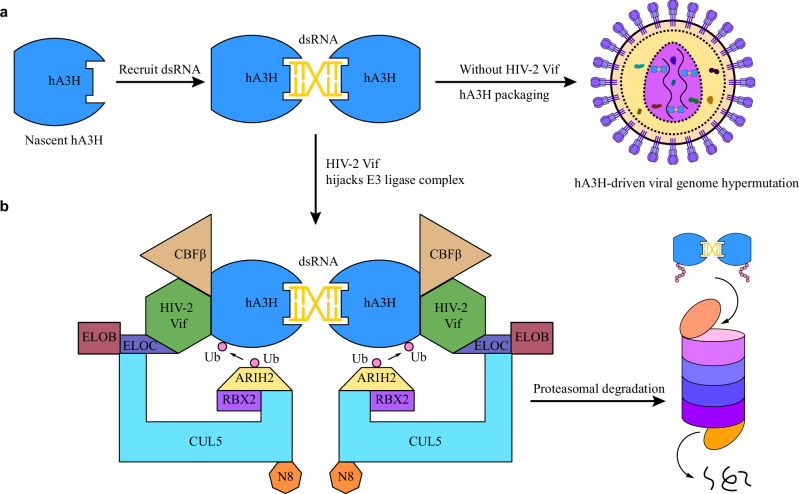
Model of HIV-2 Vif-mediated antagonism of hA3H. **a** In the absence of HIV-2 Vif, hA3H forms a dsRNA-mediated dimer and is packaged into HIV-2 virions, restricting infection through hypermutation of the viral genome. **b** In the presence of HIV-2 Vif, Vif recruits the host Cullin-RING E3 ligase complex and CBFβ, binds to both hA3H monomers, and induces hA3H polyubiquitination and proteasomal degradation. The figure was prepared using Adobe Illustrator.

The dimer organization of the HIV-2 Vif-A3H complex differs markedly from that observed in the HIV-1 Vif-cpzA3H structure (Fig. 5)^49^. In our complex, RNA-mediated and Vif-Vif interactions, previously identified as critical dimer interfaces in HIV-1 Vif-cpzA3H are not observed^49^. Moreover, both A3H monomers in the HIV-2 Vif complex are positioned to be ubiquitinated and targeted for degradation, whereas in the HIV-1 Vif-cpzA3H complex, only the proximal A3H monomer is accessible for ubiquitination (Fig. 5)^49^. These structural distinctions reveal that HIV-1 and HIV-2 Vif employ different dimerization modes and degradation mechanisms to antagonize A3H.

## Discussion

Our cryo-EM structure of the HIV-2 Vif-A3H complex, together with biochemical assays, provides a comprehensive model for the potent antagonism of human A3H by HIV-2 Vif. The structure reveals that A3H forms an RNA-mediated dimer, while HIV-2 Vif and CBFβ form a platform that engages one A3H monomer for ubiquitination and degradation. Both A3H monomers in the complex are positioned for ubiquitination, highlighting an efficient degradation mechanism (Fig.5). The dimerization of A3H in our structure is mediated solely by dsRNA, distinct from the RNA-Vif and Vif-Vif interactions reported in the HIV-1 Vif-cpzA3H complex, or the A3G-A3G and A3G-Vif interfaces observed in the HIV-1 Vif-A3G complex^44,49^. These observations suggest that RNA-mediated dimerization is a conserved feature of Vif-mediated A3 degradation, while the mode of dimer assembly varies according to the specific A3 target and Vif sequence. Interestingly, dsRNA plays distinct roles in different Vif-A3 complexes. In the HIV-2 Vif-A3H structure, dsRNA mediates A3H dimerization but does not stabilize the Vif-A3H interface. In contrast, in the HIV-1 Vif-A3H complex, RNA contributes directly to stabilizing the interaction^49^. This difference underscores the importance of nucleic acid binding in modulating Vif-A3 interactions and may reflect distinct evolutionary strategies for A3H antagonism between HIV-1 and HIV-2. In addition to its cytosine deaminase activity, A3H restricts viral replication through deaminase-independent mechanisms, including incorporation into viral particles and inhibition of reverse transcription^21,54^. RNA binding promotes A3H encapsidation and suppresses reverse transcription, suggesting that RNA-mediated A3H dimer formation represents a particularly potent antiviral state^21^. HIV-2 Vif counteracts A3H more efficiently by targeting each A3H protomer for proteasomal degradation. This mechanism may partially account for the stronger antagonistic activity of HIV-2 Vif against A3H compared with HIV-1 Vif, which mediates ubiquitination and degradation of only the proximal A3H protomer^49^.

Both HIV-2 Vif and CBFβ directly interact with A3H, stabilizing the complex sufficiently for cryo-EM analysis without crosslinking, a notable contrast to the HIV-1 Vif-A3H complex, which requires extensive mutagenesis and chemical crosslinking for structural stabilization^48,49^. The interface between HIV-2 Vif and A3H buries a larger solvent accessible surface area than that observed in HIV-1 Vif-A3H, consistent with the tighter binding affinity of the HIV-2 complex. Structural analysis identifies Vif loop3, loop5, and helix α1 as major contact regions, with a conserved Trp-rich motif in loop3 playing a central role in A3H recognition (Fig. 2). This motif is conserved in both HIV-2 Vif and smmVif but differs markedly from the corresponding region in HIV-1 and cpzVif, suggesting a conserved mechanism of A3H recognition within the HIV-2/SIVsmm lineage and divergent evolutionary trajectories for HIV-1 and HIV-2 Vif (Supplementary Fig. 8b).

Structural comparisons further emphasize the distinct binding modes of A3H with HIV-1 and HIV-2 Vif. The critical A3H residue W90, essential for HIV-1 Vif interaction, is not involved in the HIV-2 interface (Fig. 4a, b). Interestingly, A3H from sooty mangabey carries an arginine at this position, implying that diversification of SIVsmm preceded transmission to hominid primates (Supplementary Fig. 8a). Similarly, residues F39 and H48 of HIV-1 Vif, key determinants for HIV-1 Vif antagonism of A3H, are substituted with C40 and W49 in HIV-2 Vif, highlighting lineage-specific adaptations (Supplementary Fig. 8b)^19,33,55^. Together, these observations suggest that HIV-1 and HIV-2 evolved distinct structural solutions to counteract A3H restriction. One key caveat to this conclusion, however, is that the reported HIV-1 Vif-A3H structures were obtained using the NL4-3 strain, which is not optimized for hA3H degradation. Importantly, hA3H and cpzA3H differ in their intracellular localization and in their responses to primate lentiviral Vif^17,24^. Consequently, the mode of interaction between optimized HIV-1 Vif and wild-type hA3H may differ in viral strains that have naturally evolved in PLWH who are homozygous for stable A3H haplotype II^19^.

CBFβ is required for HIV and SIV Vif-mediated antagonism of A3 family members^30,56^. Structural studies of VCBC with A3G reveal that CBFβ does not contact A3G but instead plays an allosteric role in Vif-mediated degradation by stabilizing the fold of the VCBC substrate receptor module for the Cullin-5 E3 ligase^30,44–46,51,57^. In addition to this “chaperone” function of CBFβ, a prior report suggested it makes direct interactions with A3F_CTD_ when bound to HIV-1 Vif^47^. However, the generality of CBFβ playing a direct role in binding A3 family members was unknown. As observed in our structure, CBFβ directly contributes to A3H binding in the HIV-2 complex. CBFβ R52 inserts into a negatively charged pocket of A3H, enhancing complex stability (Supplementary Fig. 6a). This pocket is also involved in HIV-1 Vif interaction, suggesting that it may serve as a common protein-protein interface in the Vif-A3H complex^48,49^. Our studies of HIV-2 Vif and A3H and a prior study of HIV-1 Vif with A3F_CTD_ suggest one reason primate lentiviral Vif hijacks CBFβ is to provide additional binding surfaces to enhance the ability of Vif to counteract the expanded A3 repertoire found in primates compared with other mammals^47,58^. Additionally, we present evidence supporting a degradation-independent mechanism as reported for HIV-1 Vif antagonism of A3G^59^. For example, the CBFβ W73A mutation impairs HIV-2 Vif-induced degradation of A3H without noticeably restoring A3H packaging. (Fig. 3e). CBFβ W73 forms an aromatic cluster with W49 and W51 of HIV-2 Vif. The increased steady-state level of A3H may be due to reduced HIV-2 Vif stability caused by the CBFβ W73A mutation, which weakens its interaction with Vif. However, the elevated A3H is not incorporated into VLPs, suggesting a degradation-independent mechanism by which HIV-2 Vif inhibits A3H encapsidation.

Structural comparison of our complex with HIV-1 Vif complexes bound to A3G and A3F_CTD_ revealed that Vif engages A3 proteins through a broadly overlapping interface (Supplementary Fig. 10)^44,47^. Although A3H contains only a single cytidine deaminase (CDA) domain, its fold closely resembles the CDA domains of A3G and A3F_CTD_ (Supplementary Fig. 10a–d). In the HIV-2 Vif-CBFβ-A3H complex, the A3H CDA domain occupies the space between the two CDA domains of A3G, thereby establishing a distinct binding mode (Supplementary Fig. 10e). This domain partially overlaps with the position of A3F_CTD_ but requires rotation to align its interaction surface with Vif (Supplementary Fig. 10f). The buried solvent-accessible surface area of the Vif-CBFβ interface with A3H (1,354.78 Å²) and A3F_CTD_ (1,004 Å²) is substantially larger than that with A3G (560 Å²), supporting tight protein-protein interactions in the former and RNA-dependent stabilization in the latter. Alignment of Vif proteins showed that HIV-1 Vif exploits an interface composed of loop3, loop5, and helix α1 to target A3G and A3F_CTD_ (Supplementary Fig. 10g). Similarly, HIV-2 Vif utilizes the same surface to target A3H hapII (Supplementary Fig. 10g). However, the interactions between HIV-2 Vif and other A3 family members require further investigation.

In summary, our structural and biochemical analyses provide a detailed framework for understanding HIV-2 Vif antagonism of stable human A3H. HIV-2 Vif engages A3H through a distinct interface involving cooperative binding with CBFβ and dsRNA-mediated A3H dimerization. These findings reveal mechanistic divergence between HIV-1 and HIV-2 in their strategies to evade host restriction and provide insights into the molecular basis of HIV-2’s unique evolutionary trajectory. Moreover, approximately 2 million people worldwide are infected with HIV-2, which is intrinsically resistant to non-nucleoside reverse transcriptase inhibitors and shows reduced susceptibility to some protease inhibitors used for HIV-1 treatment^60^. Therapeutic options for HIV-2 are therefore more limited than for HIV-1. Consistent with this, lenacapavir, the first approved capsid inhibitor for HIV-1, exhibits over 10-fold lower potency against HIV-2, with most patients developing capsid-associated drug resistance mutations^61,62^. This work highlights potential avenues for targeting Vif-A3 interactions in the development of antiviral therapeutics.

## Methods

### Cell lines

HEK293T, HEK293T/17, and HEK293T-shCBFβ cells were maintained in Dulbecco’s modified Eagle’s medium (DMEM) supplemented with 10% fetal bovine serum (FBS) at 37 °C in a humidified incubator with 5% CO₂. SupT1 cells were cultured in RPMI 1640 medium containing 10% FBS, 1% Antibiotic-Antimycotic, 1% sodium pyruvate, 1% glucose, and 1% GlutaMAX under the same conditions. Sf9 and Hi5 insect cells were maintained in Sf-900 III SFM medium at 27 °C with shaking at 120 rpm and used for recombinant baculovirus production and protein expression.

### Protein expression and purification

Six HIV-2 Vif sequences representing both major HIV-2 subtypes and spanning broad sequence diversity were codon-optimized and screened for expression in insect cells. HIV-2 Vif from ALI strain (Accession number: AF082339), which was isolated from people living with HIV, encodes an amino acid sequence closely matching the clade A consensus. The wild-type ALI Vif exhibited high expression in insect cells and robust activity against A3H hapII, and was therefore selected for structural study. Wild-type human A3H hapII fused to a C-terminal 2xStrep tag, HIV-2 Vif (ALI strain, wild type), CBFβ, and subunits of the Cullin-RING E3 ligase complex (ELOB, ELOC, and CUL5 residues 1–386, referred to as CUL5N) were cloned into a single MacroBac vector using a restriction-ligation strategy as previously described^63^. Baculoviruses were generated with the Bac-to-Bac system and amplified in Sf9 insect cells. Optimal virus titers for protein expression in Hi5 cells (2.0 x 10⁶ cells ml⁻¹) were determined by serial dilution and analyzed by immunoblot. Large-scale protein expression was performed in Hi5 cells infected with the optimized virus titer. Cells were harvested 48 h post-infection by centrifugation at 1500 × *g* for 10 min, washed once with PBS, flash-frozen in liquid nitrogen, and stored at −80 °C until use.

Cell pellets from 1 L of Hi5 culture were resuspended in 50 mL lysis buffer containing 50 mM HEPES (pH 8.0), 50 mM NaCl, 5% (v/v) glycerol, 1% (v/v) Triton X-100, 5 mM MgCl₂, 5 mM CaCl₂, 1 mM TCEP, cOmplete protease inhibitor tablets (Sigma-Aldrich, 5 tablets), 25 µg mL^−1^ DNase I, and 50 µg mL^−1^ RNase A. Cells were lysed using a Dounce homogenizer and incubated on ice for 30 min before centrifugation at 17,000 × *g* for 1 h at 4 °C to remove debris. The clarified lysate was loaded onto a 4 mL Strep-Tactin XT Sepharose column (Cytiva) pre-equilibrated with binding buffer (50 mM HEPES pH 8.0, 150 mM NaCl, 5% glycerol, 1 mM TCEP). The column was washed sequentially with 50 mL wash buffer (50 mM HEPES pH 8.0, 1.5 M NaCl, 5% glycerol, 1 mM TCEP) and 50 mL binding buffer. On-column RNase A digestion was performed by incubating with 20 mL binding buffer supplemented with 50 µg mL^−1^ RNase A overnight at 4 °C. The column was washed with binding buffer to remove residual RNase A, and the complex was eluted with elution buffer (50 mM HEPES, pH 8.0, 150 mM NaCl, 5% glycerol, 1 mM TCEP, 50 mM biotin). The Strep-Tactin eluate was diluted four-fold with buffer A (50 mM HEPES pH 7.0, 75 mM NaCl, 5% glycerol, 1 mM TCEP) and loaded onto a 5 mL HiTrap Heparin HP column (GE Healthcare) pre-equilibrated with the same buffer. The complex was eluted using a linear NaCl gradient from 75 mM to 1 M. Fractions containing all six components were pooled and further purified by size-exclusion chromatography on a Superose 6 Increase 10/300 GL column (GE Healthcare) equilibrated with SEC buffer (30 mM HEPES pH 7.0, 150 mM NaCl, 5% glycerol, 1 mM TCEP). The peak fraction was diluted and used for cryo-EM sample preparation.

### Cryo-EM grid preparation and data collection

The purified HIV-2 Vif-A3H complex was diluted to an A_280_ of 0.75, corresponding to an estimated concentration of 4.1 µM. Aliquots of 3 µL were applied to glow-discharged Quantifoil Au 300 mesh R1.2/1.3 grids (Electron Microscopy Sciences). Grids were blotted for 2 s with a blot force of 1 at 4 °C and 100% humidity using a Thermo Fisher Vitrobot Mark IV, then plunge-frozen in liquid ethane cooled by liquid nitrogen.

Cryo-grids were initially screened on a Thermo Fisher Arctica microscope operated at 200 kV, and high-quality grids were subsequently transferred to a Titan Krios microscope (Thermo Fisher Scientific) operated at 300 kV for data collection. Movies were recorded on a Gatan K3 direct electron detector mounted post a quantum energy filter (20 eV slit width) in super-resolution mode at a calibrated pixel size of 0.4094 Å, at a nominal magnification of ×105,000. Data were collected semi-automatically using SerialEM with defocus values ranging from −1.2 µm to −2.5 µm, a dose rate of 16 e^−^/pixel/s, and an exposure time of 2 s (0.025 s per frame), yielding 80 frames per stack and a total dose of 47.7 e^−^/Å²^64^. Movie stacks were motion-corrected using MotionCor2 within the SCIPION framework and binned twofold, resulting in a physical pixel size of 0.8189 Å per pixel^65,66^.

### Cryo-EM image processing and model building

Dose-weighted, motion-corrected micrographs were imported into cryoSPARC v4.7.0 for further processing^67^. Contrast transfer function (CTF) parameters were estimated using Patch CTF estimation, and micrographs with CTF resolutions worse than 5 Å or with excessive ice contamination were manually excluded. A total of 6,469,148 particles were automatically picked from 9765 micrographs using reference-free Blob Picker. Particles were extracted in a 360-pixel box and binned fourfold to 90 pixels (3.2756 Å per pixel). Two rounds of 2D classification were performed to remove ice, contaminants, and aggregates. A subset of 100,000 particles selected from 870,536 high-quality particles was used for ab initio reconstruction into four classes. Particles from the 2D classification were then re-extracted without binning and subjected to heterogeneous refinement using the generated references. The best class, containing 404,870 particles, was further refined using Non-uniform Refinement and Local Refinement, yielding a 3.84 Å reconstruction. To improve particle recovery and map quality, the 3.84 Å reconstruction was low-pass filtered to 20 Å and used as a template for additional particle picking using Template Picker. A total of 8,192,985 particles were picked and extracted from the original micrographs and subjected to two rounds of heterogeneous refinement without 2D classification. The first round used one good reference and four junk references, yielding 2,890,590 particles. In the second round, references were generated by low-pass filtering the 3.84 Å map to 15, 30, 45, and 60 Å, resulting in 1,257,210 particles in the best class. These particles underwent Non-uniform Refinement and Local Refinement with a mask generated in cryoSPARC, yielding a 3.21 Å reconstruction corresponding to the HIV-2 Vif-A3H dimer. To further improve the density of the HIV-2 Vif-A3H monomer, a monomer-specific mask was generated for an additional round of heterogeneous refinement. The resulting 545,960 particles produced a 3.26 Å map after Non-uniform Refinement and Local Refinement, using the gold-standard FSC = 0.143 criterion^68^.

Initial models of hA3H-dsRNA and the HIV-2 Vif-CBFβ subcomplex were predicted using AlphaFold3^69^. The models were docked into the cryo-EM density map in UCSF Chimera and manually rebuilt in Coot^70,71^. The HIV-2 Vif-A3H monomer model was refined using the Phenix.real_space_refine module in Phenix with secondary structure and geometry restraints applied^72,73^. Protein main chains and side-chain rotamers were manually adjusted in Coot, followed by iterative cycles of real-space refinement in Phenix and manual rebuilding. The final model was validated using the comprehensive cryo-EM validation tool in Phenix^72^. The refined HIV-2 Vif-A3H monomer model served as a template for dimer model building, which was refined and validated following the same procedure. All structural figures were prepared using UCSF ChimeraX or PyMOL (Schrodinger LLC)^74^.

### Co-immunoprecipitation and Immunoblot

Full-length HIV-2 Vif fused with a C-terminal 3xFLAG tag, full-length CBFβ with an N-terminal Myc tag, and full-length human A3H fused with a C-terminal HA tag were cloned into the pcDNA3.1 expression vector using a seamless assembly method. Site-directed mutagenesis was performed using PrimeSTAR MAX DNA Polymerase (Takara) according to the manufacturer’s instructions. The primer sets used for mutagenesis are listed in Supplementary Table 2. All constructs were verified by whole-plasmid sequencing.

For analysis of the HIV-2 Vif-A3H interaction, HEK293T cells were transfected separately with 2 µg of HIV-2 Vif-3xFLAG (WT or mutants) and 2 µg of A3H-HA in 10-cm dishes using TransIT-LT1 Transfection Reagent (Mirus Bio). For analysis of the CBFβ-A3H interaction, HEK293T-shCBFβ cells were co-transfected with 1 µg of CBFβ-Myc (WT or mutants) and 1 µg of HIV-2 Vif-3xFLAG to express the Vif-CBFβ complex, and A3H-HA (2 µg) was transfected separately into HEK293T-shCBFβ cells. Cells were harvested 48 h post-transfection and lysed in buffer containing 50 mM HEPES (pH 8.0), 150 mM NaCl, 5% (v/v) glycerol, 1% (v/v) Triton X-100, cOmplete Protease Inhibitor, and 50 µg/mL RNase A.

For co-immunoprecipitation, clarified cell lysates expressing HIV-2 Vif alone or HIV-2 Vif-CBFβ complex were combined with lysates expressing A3H and incubated with Anti-FLAG M2 Magnetic Beads for 2 h at 4 °C. Input samples were collected prior to incubation with the beads. The beads were washed five times with wash buffer (50 mM HEPES, pH 8.0, 150 mM NaCl, and 5% glycerol), and bound proteins were competitively eluted with 3xFLAG peptide (100 µg/mL). Immunoprecipitated samples were separated by SDS-PAGE using 4–15% Precast Protein Gels and transferred onto nitrocellulose membranes with the Trans-Blot Turbo system (Bio-Rad). Membranes were probed with mouse anti-FLAG M2 (Sigma-Aldrich, F3165, 1:5000), rabbit anti-HA (Cell Signaling Technology, 3724, 1:5000), and mouse anti-GAPDH (Proteintech Group, 60004-1-Ig, 1:10,000) antibodies, followed by StarBright Blue 520 Goat Anti-Rabbit IgG (Bio-Rad, 12005869, 1:5000) and StarBright Blue 700 Goat Anti-Mouse IgG (Bio-Rad, 12004158, 1:5000) secondary antibodies. Fluorescent signals were detected using a ChemiDoc MP Imaging System (Bio-Rad).

### A3H packaging and vif-mediated degradation assays

To analyze A3H packaging affected by HIV-2 Vif mutants, HEK293T cells (1.5 × 10⁵ cells/mL) seeded in 6-well plates were co-transfected with pLAIΔenvLuc2Δvif (500 ng) and A3H-HA (100 ng) plasmids, with or without HIV-2 Vif-3xFLAG (50 ng), using TransIT-LT1 Transfection Reagent (Mirus Bio). For analysis of CBFβ mutant effects, HEK293T-shCBFβ cells (1.5 × 10⁵ cells/mL) seeded in 6-well plates were co-transfected with pLAIΔenvLuc2Δvif (500 ng), A3H-HA (100 ng), and CBFβ-Myc (100 ng), either in the absence or presence of HIV-2 Vif-3xFLAG (50 ng), using TransIT-LT1 Transfection Reagent (Mirus Bio). Twenty-four hours after transfection, the medium was replaced with fresh DMEM supplemented with 10% FBS. Viral-like particles (VLPs) were harvested 72 h post-transfection by filtering the supernatant through 0.22 μm syringe filters, followed by centrifugation at 20,160 × *g* for 30 min at 4 °C. The resulting pellets containing VLPs were resuspended in PBS and analyzed by SDS-PAGE. A3H packaging into VLPs was examined by immunoblot using anti-HA (Cell Signaling Technology, 3724, 1:5000) and anti-p24gag antibodies (BEI Resources, 3537, 1:5000), as previously described.

For Vif-mediated degradation assays, transfected cells were harvested 72 h post-transfection and lysed in buffer containing 50 mM HEPES (pH 8.0), 150 mM NaCl, 5% (v/v) glycerol, 1% (v/v) Triton X-100, and cOmplete Protease Inhibitor Cocktail. Cell lysates were subjected to immunoblot analysis using anti-FLAG (Sigma-Aldrich, F3165, 1:5000), anti-HA (Cell Signaling Technology, 3724, 1:5000), and anti-GAPDH (Proteintech Group, 60004-1-Ig, 1:10,000) antibodies to assess steady-state expression levels of A3H and HIV-2 Vif.

### Viral infectivity assay

Single cycle infectivity assays were performed as previously described^75^. Untagged WT or mutant of the HIV-2 Vif sequences were cloned into the pLAIΔenvLuc2ΔVif proviral vector. HEK293T/17 cells were seeded at 1.5 × 10^5^ cells/mL in 6-well plates and transfected with 400 ng of pLAIΔenvLuc2 ALI Vif proviral vector, 100 ng of L-VSV-G, and 200 ng of HA-tagged hA3H using TransIT-LT1 transfection reagent at a ratio of 3.5 µL reagent per µg DNA. For transfections in HEK293T-shCBFβ cells, an additional 100 ng of CBFβ expression plasmid was included. At 24 h post-transfection, media were replaced. Viral supernatants were harvested at 72 h post-transfection and clarified by filtration through 0.22-µm PES filters. Viral titers were quantified by RT-qPCR based titration^76^. Briefly, viral supernatants mixed 1:1 with lysis buffer containing RNase inhibitors (RnaseOut, Thermofisher, 10777019). Samples were incubated for 10 min at room temperature, then diluted 10-fold in H_2_O, and used as the input for the assay. Samples were then combined with reaction buffer consisting Rox SYBR MasterMix dTTP Blue (Takyon, UF-RSMT-B0701), RNase inhibitors, MS2 RNA (Roche, 10165948001), and published primers for reverse transcription and amplification. Melting peaks were automatically calculated by QuantStudio Design and Analysis software. A standard curve generated from replication-competent HIV-1 supernatants with known reverse transcriptase activity was included in parallel in each assay. Reverse transcription activity was calculated from the mean of four technical qPCR replicates. Equal infectious units were used for all infections, with mock-transfected supernatant added as needed to equalize final volumes.

SupT1 cells were seeded at 1.5 × 10^5^ cells/mL in U-bottom 96-well plates in media supplemented with 20 µg/mL DEAE-dextran and infected by spinoculation at 1100 × *g* for 30 min at 30 °C. All infections were performed in technical duplicates, and each condition was tested in three independent biological replicates. At 72 h post-infection, cells were lysed with 100 µL Bright-Glo reagent (Promega, E2620), and luminescence was measured using a LUMIstar Omega luminometer (BMG Labtech). The percent infectivity was calculated by normalizing the relative light units (RLUs) from each biological replicate containing A3H protein to the mean RLUs of the corresponding virus produced in the absence of A3H.

### Reporting summary

Further information on research design is available in the Nature Portfolio Reporting Summary linked to this article.

## Supplementary information

Supplementary Information

Description of Additional Supplementary Files

Supplementary Movie 1

Reporting Summary

Transparent Peer Review file

## Source data

Source Data

## Data Availability

The cryo-EM maps and atomic models of the HIV-2 Vif-A3H-CBFβ complex generated in this study have been deposited in the Electron Microscopy Data Bank (EMDB) and the Protein Data Bank (PDB). The monomeric complex is available under accession codes EMDB: EMD-74939 and PDB: 9ZY0, and the dimeric complex under EMDB: EMD-74940 and PDB: 9ZY1. PDB entries (8FVI, 5W3V, 4N9F, 7ONI, 7B5M, 8CX0, 6NIL) used in this study were downloaded from Protein Data Bank. Source data are provided with this paper.

## References

[1] Delviks-Frankenberry, K. A., Desimmie, B. A. & Pathak, V. K. Structural insights into APOBEC3-mediated lentiviral restriction. *Viruses* **12**, 10.3390/v12060587 (2020). 10.3390/v12060587 PMC7354603 32471198

[2] Feng, Y., Baig, T. T., Love, R. P. & Chelico, L. Suppression of APOBEC3-mediated restriction of HIV-1 by Vif. *Front. Microbiol.* **5**, 450 (2014). 25206352 10.3389/fmicb.2014.00450 PMC4144255

[3] Li, Y. L., Langley, C. A., Emerman, M. & Gross, J. D. APOBEC3G antagonism by Vif, or when structure meets biological and evolutionary studies. *Annu. Rev. Virol.* **12**, 451–469 (2025). 40198850 10.1146/annurev-virology-092623-091351 PMC13016362

[4] Harris, R. S. & Dudley, J. P. APOBECs and virus restriction. *Virology* **479-480**, 131–145 (2015). 25818029 10.1016/j.virol.2015.03.012 PMC4424171

[5] Mariani, R. et al. Species-specific exclusion of APOBEC3G from HIV-1 virions by Vif. *Cell* **114**, 21–31 (2003). 12859895 10.1016/s0092-8674(03)00515-4

[6] Zhang, H. et al. The cytidine deaminase CEM15 induces hypermutation in newly synthesized HIV-1 DNA. *Nature* **424**, 94–98 (2003). 12808465 10.1038/nature01707 PMC1350966

[7] Lecossier, D., Bouchonnet, F., Clavel, F. & Hance, A. J. Hypermutation of HIV-1 DNA in the absence of the Vif protein. *Science* **300**, 1112 (2003). 12750511 10.1126/science.1083338

[8] Mangeat, B. et al. Broad antiretroviral defence by human APOBEC3G through lethal editing of nascent reverse transcripts. *Nature* **424**, 99–103 (2003). 12808466 10.1038/nature01709

[9] York, A., Kutluay, S. B., Errando, M. & Bieniasz, P. D. The RNA binding specificity of human APOBEC3 proteins resembles that of HIV-1 nucleocapsid. *PLoS Pathog.* **12**, ARTN e100583310 (2016). 10.1371/journal.ppat.1005833 PMC4991800 27541140

[10] Huthoff, H., Autore, F., Gallois-Montbrun, S., Fraternali, F. & Malim, M. H. RNA-dependent oligomerization of APOBEC3G is required for restriction of HIV-1. *PLoS Pathog.* **5**, ARTN e100033010 (2009). 10.1371/journal.ppat.1000330 PMC2646141 19266078

[11] Mohammadzadeh, N. et al. Role of co-expressed APOBEC3F and APOBEC3G in inducing HIV-1 drug resistance. *Heliyon* **5**, e01498 (2019). 31025011 10.1016/j.heliyon.2019.e01498 PMC6475876

[12] Hultquist, J. F. et al. Human and rhesus APOBEC3D, APOBEC3F, APOBEC3G, and APOBEC3H demonstrate a conserved capacity to restrict Vif-deficient HIV-1. *J. Virol.* **85**, 11220–11234 (2011). 21835787 10.1128/JVI.05238-11 PMC3194973

[13] Olson, M. E., Harris, R. S. & Harki, D. A. APOBEC enzymes as targets for virus and cancer therapy. *Cell Chem. Biol.* **25**, 36–49 (2018). 29153851 10.1016/j.chembiol.2017.10.007 PMC5775913

[14] OhAinle, M., Kerns, J. A., Li, M. M. H., Malik, H. S. & Emerman, M. Antiretroelement activity of APOBEC3H was lost twice in recent human evolution. *Cell Host Microbe* **4**, 249–259 (2008). 18779051 10.1016/j.chom.2008.07.005 PMC2608726

[15] Dang, Y. et al. Human cytidine deaminase APOBEC3H restricts HIV-1 replication. *J. Biol. Chem.* **283**, 11606–11614 (2008). 18299330 10.1074/jbc.M707586200 PMC2430661

[16] Feng, Y. Q. et al. Natural polymorphisms and oligomerization of human APOBEC3H contribute to single-stranded DNA scanning ability. *J. Biol. Chem.* **290**, 27188–27203 (2015). 26396192 10.1074/jbc.M115.666065 PMC4646388

[17] Li, M. M. & Emerman, M. Polymorphism in human APOBEC3H affects a phenotype dominant for subcellular localization and antiviral activity. *J. Virol.* **85**, 8197–8207 (2011). 21653666 10.1128/JVI.00624-11 PMC3147987

[18] Chesarino, N. M. & Emerman, M. Polymorphisms in human APOBEC3H differentially regulate ubiquitination and antiviral activity. *Viruses* **12**, 10.3390/v12040378 (2020). 10.3390/v12040378 PMC7232234 32235597

[19] Refsland, E. W. et al. Natural polymorphisms in human APOBEC3H and HIV-1 Vif combine in primary T lymphocytes to affect viral G-to-A mutation levels and infectivity. *PLoS Genet.* **10**, e1004761 (2014). 25411794 10.1371/journal.pgen.1004761 PMC4238949

[20] Ito, F. et al. Understanding the structure, multimerization, subcellular localization and mC selectivity of a genomic mutator and anti-HIV factor APOBEC3H. *Sci. Rep.* **8**, 3763 (2018). 29491387 10.1038/s41598-018-21955-0 PMC5830531

[21] Shaban, N. M. et al. The antiviral and cancer genomic DNA deaminase APOBEC3H is regulated by an RNA-mediated dimerization mechanism. *Mol. Cell* **69**, 75–86.e9 (2018). 29290613 10.1016/j.molcel.2017.12.010 PMC5991973

[22] Matsuoka, T. et al. Structural basis of chimpanzee APOBEC3H dimerization stabilized by double-stranded RNA. *Nucleic Acids Res.* **46**, 10368–10379 (2018). 30060196 10.1093/nar/gky676 PMC6212771

[23] Bohn, J. A. et al. APOBEC3H structure reveals an unusual mechanism of interaction with duplex RNA. *Nat. Commun.* **8**, 1021 (2017). 29044109 10.1038/s41467-017-01309-6 PMC5647330

[24] Zhang, Z. et al. Stably expressed APOBEC3H forms a barrier for cross-species transmission of simian immunodeficiency virus of chimpanzee to humans. *PLoS Pathog.* **13**, e1006746 (2017). 29267382 10.1371/journal.ppat.1006746 PMC5739507

[25] Li, M. M., Wu, L. I. & Emerman, M. The range of human APOBEC3H sensitivity to lentiviral Vif proteins. *J. Virol.* **84**, 88–95 (2010). 19828612 10.1128/JVI.01344-09 PMC2798431

[26] Sheehy, A. M., Gaddis, N. C., Choi, J. D. & Malim, M. H. Isolation of a human gene that inhibits HIV-1 infection and is suppressed by the viral Vif protein. *Nature* **418**, 646–650 (2002). 12167863 10.1038/nature00939

[27] Yu, X. et al. Induction of APOBEC3G ubiquitination and degradation by an HIV-1 Vif-Cul5-SCF complex. *Science* **302**, 1056–1060 (2003). 14564014 10.1126/science.1089591

[28] Marin, M., Rose, K. M., Kozak, S. L. & Kabat, D. HIV-1 Vif protein binds the editing enzyme APOBEC3G and induces its degradation. *Nat. Med.* **9**, 1398–1403 (2003). 14528301 10.1038/nm946

[29] Sheehy, A. M., Gaddis, N. C. & Malim, M. H. The antiretroviral enzyme APOBEC3G is degraded by the proteasome in response to HIV-1 Vif. *Nat. Med.* **9**, 1404–1407 (2003). 14528300 10.1038/nm945

[30] Jager, S. et al. Vif hijacks CBF-beta to degrade APOBEC3G and promote HIV-1 infection. *Nature* **481**, 371–375 (2011). 22190037 10.1038/nature10693 PMC3310910

[31] Binka, M., Ooms, M., Steward, M. & Simon, V. The activity spectrum of Vif from multiple HIV-1 subtypes against APOBEC3G, APOBEC3F, and APOBEC3H. *J. Virol.* **86**, 49–59 (2012). 22013041 10.1128/JVI.06082-11 PMC3255910

[32] Zhang, W., Du, J., Evans, S. L., Yu, Y. & Yu, X. F. T-cell differentiation factor CBF-beta regulates HIV-1 Vif-mediated evasion of host restriction. *Nature* **481**, 376–379 (2011). 22190036 10.1038/nature10718

[33] Ooms, M., Letko, M., Binka, M. & Simon, V. The resistance of human APOBEC3H to HIV-1 NL4-3 molecular clone is determined by a single amino acid in Vif. *PLoS ONE* **8**, ARTN e5774410 (2013). 10.1371/journal.pone.0057744 PMC3585137 23469063

[34] Smith, J. L., Izumi, T., Borbet, T. C., Hagedorn, A. N. & Pathak, V. K. HIV-1 and HIV-2 Vif interact with human APOBEC3 proteins using completely different determinants. *J. Virol.* **88**, 9893–9908 (2014). 24942576 10.1128/JVI.01318-14 PMC4136346

[35] Nchioua, R. et al. APOBEC3F constitutes a barrier to successful cross-species transmission of simian immunodeficiency virus SIVsmm to humans. *J. Virol.* **95**, e0080821 (2021). 34132575 10.1128/JVI.00808-21 PMC8354236

[36] Bailes, E. et al. Hybrid origin of SIV in chimpanzees. *Science* **300**, 1713 (2003). 12805540 10.1126/science.1080657

[37] Rambaut, A., Posada, D., Crandall, K. A. & Holmes, E. C. The causes and consequences of HIV evolution. *Nat. Rev. Genet.* **5**, 52–61 (2004). 14708016 10.1038/nrg1246

[38] Heeney, J. L., Dalgleish, A. G. & Weiss, R. A. Origins of HIV and the evolution of resistance to AIDS. *Science* **313**, 462–466 (2006). 16873637 10.1126/science.1123016

[39] Binning, J. M., Chesarino, N. M., Emerman, M. & Gross, J. D. Structural basis for a species-specific determinant of an SIV Vif protein toward hominid APOBEC3G antagonism. *Cell Host Microbe* **26**, 739–747 e734 (2019). 31830442 10.1016/j.chom.2019.10.014 PMC6913891

[40] Hahn, B. H., Shaw, G. M., De Cock, K. M. & Sharp, P. M. AIDS - AIDS as a zoonosis: scientific and public health implications. *Science* **287**, 607–614 (2000). 10649986 10.1126/science.287.5453.607

[41] Etienne, L., Hahn, B. H., Sharp, P. M., Matsen, F. A. & Emerman, M. Gene loss and adaptation to hominids underlie the ancient origin of HIV-1. *Cell Host Microbe* **14**, 85–92 (2013). 23870316 10.1016/j.chom.2013.06.002 PMC3733229

[42] Hirsch, V. M., Olmsted, R. A., Murphey-Corb, M., Purcell, R. H. & Johnson, P. R. An African primate lentivirus (SIVsm) closely related to HIV-2. *Nature* **339**, 389–392 (1989). 2786147 10.1038/339389a0

[43] Compton, A. A. & Emerman, M. Convergence and divergence in the evolution of the APOBEC3G-Vif interaction reveal ancient origins of simian immunodeficiency viruses. *PLoS Pathog.* **9**, e1003135 (2013). 23359341 10.1371/journal.ppat.1003135 PMC3554591

[44] Li, Y. L. et al. The structural basis for HIV-1 Vif antagonism of human APOBEC3G. *Nature* **615**, 728–733 (2023). 36754086 10.1038/s41586-023-05779-1 PMC10033410

[45] Kouno, T. et al. Structural insights into RNA bridging between HIV-1 Vif and antiviral factor APOBEC3G. *Nat Commun* **14**, 10.1038/s41467-023-39796-5 (2023). 10.1038/s41467-023-39796-5 PMC10328928 37419875

[46] Ito, F. et al. Structural basis for HIV-1 antagonism of host APOBEC3G via Cullin E3 ligase. *Sci. Adv.* **9**, eade3168 (2023). 36598981 10.1126/sciadv.ade3168 PMC9812381

[47] Hu, Y. et al. Structural basis of antagonism of human APOBEC3F by HIV-1 Vif. *Nat. Struct. Mol. Biol.* **26**, 1176–1183 (2019). 31792451 10.1038/s41594-019-0343-6 PMC6899190

[48] Ito, F., Alvarez-Cabrera, A. L., Kim, K., Zhou, Z. H. & Chen, X. S. Structural basis of HIV-1 Vif-mediated E3 ligase targeting of host APOBEC3H. *Nat. Commun.* **14**, 5241 (2023). 37640699 10.1038/s41467-023-40955-x PMC10462622

[49] Skorupka, K. A. et al. HIV-1 vif mediates ubiquitination of the proximal protomer in the APOBEC3H dimer to induce degradation. *Nat. Commun.* **16**, Artn 587910 (2025). 10.1038/s41467-025-60984-y PMC12217271 40593686

[50] Pickart, C. M. Mechanisms underlying ubiquitination. *Annu. Rev. Biochem.* **70**, 503–533 (2001). 11395416 10.1146/annurev.biochem.70.1.503

[51] Guo, Y. Y. et al. Structural basis for hijacking CBF-β and CUL5 E3 ligase complex by HIV-1 Vif. *Nature* **505**, 229–233 (2014). 24402281 10.1038/nature12884

[52] Kostrhon, S. et al. CUL5-ARIH2 E3-E3 ubiquitin ligase structure reveals cullin-specific NEDD8 activation. *Nat. Chem. Biol.* **17**, 1075–1083 (2021). 34518685 10.1038/s41589-021-00858-8 PMC8460447

[53] Horn-Ghetko, D. et al. Ubiquitin ligation to F-box protein targets by SCF-RBR E3-E3 super-assembly. *Nature* **590**, 671–676 (2021). 33536622 10.1038/s41586-021-03197-9 PMC7904520

[54] Bohn, J. A. et al. Flexibility in nucleic acid binding is central to APOBEC3H antiviral activity. *J. Virol.* **93**, ARTN e01275–ARTN e01910 (2019). 10.1128/JVI.01275-19 PMC6880157 31578294

[55] Ooms, M. et al. HIV-1 Vif adaptation to human APOBEC3H haplotypes. *Cell Host Microbe* **14**, 411–421 (2013). 24139399 10.1016/j.chom.2013.09.006

[56] Hultquist, J. F., Binka, M., LaRue, R. S., Simon, V. & Harris, R. S. Vif proteins of human and simian immunodeficiency viruses require cellular CBFβ to degrade APOBEC3 restriction factors. *J. Virol.* **86**, 2874–2877 (2012). 22205746 10.1128/JVI.06950-11 PMC3302251

[57] Kim, D. Y. et al. CBFβ stabilizes HIV Vif to counteract APOBEC3 at the expense of RUNX1 target gene expression. *Mol. Cell* **49**, 632–644 (2013). 23333304 10.1016/j.molcel.2012.12.012 PMC3582769

[58] Kane, J. R. et al. Lineage-specific viral hijacking of non-canonical E3 ubiquitin ligase cofactors in the evolution of Vif Anti-APOBEC3 activity. *Cell Rep.* **11**, 1236–1250 (2015). 25981045 10.1016/j.celrep.2015.04.038 PMC4613747

[59] Stupfler, B., Verriez, C., Gallois-Montbrun, S., Marquet, R. & Paillart, J. C. Degradation-independent inhibition of APOBEC3G by the HIV-1 Vif protein. *Viruses* **13**, 10.3390/v13040617 (2021). 10.3390/v13040617 PMC8066197 33916704

[60] Ceccarelli, G. et al. Human immunodeficiency virus type 2: the neglected threat. *Pathogens* **10**, 10.3390/pathogens10111377 (2021). 10.3390/pathogens10111377 PMC8621479 34832533

[61] Smith, R. A. et al. Antiviral activity of lenacapavir against human immunodeficiency virus type 2 (HIV-2) isolates and drug-resistant HIV-2 mutants. *J. Infect. Dis.* **229**, 1290–1294 (2024). 38060982 10.1093/infdis/jiad562 PMC11095534

[62] Montrouge, T. et al. Rapid selection of HIV-2 capsid mutations in salvage therapy with lenacapavir-containing regimen. *Clin. Infect. Dis.* **81**, 341–344 (2025). 39737782 10.1093/cid/ciae650

[63] Gradia, S. D. et al. MacroBac: new technologies for robust and efficient large-scale production of recombinant multiprotein complexes. *Methods Enzymol.* **592**, 1–26 (2017). 28668116 10.1016/bs.mie.2017.03.008 PMC6028233

[64] Mastronarde, D. N. SerialEM: a program for automated tilt series acquisition on Tecnai microscopes using prediction of specimen position. *Microsc. Microanal.* **9**, 1182–1183 (2003).

[65] de la Rosa-Trevin, J. M. et al. Scipion: a software framework toward integration, reproducibility and validation in 3D electron microscopy. *J. Struct. Biol.* **195**, 93–99 (2016). 27108186 10.1016/j.jsb.2016.04.010

[66] Zheng, S. Q. et al. MotionCor2: anisotropic correction of beam-induced motion for improved cryo-electron microscopy. *Nat. Methods* **14**, 331–332 (2017). 28250466 10.1038/nmeth.4193 PMC5494038

[67] Punjani, A., Rubinstein, J. L., Fleet, D. J. & Brubaker, M. A. CryoSPARC: algorithms for rapid unsupervised cryo-EM structure determination. *Nat. Methods* **14**, 290–296 (2017). 28165473 10.1038/nmeth.4169

[68] Chen, S. et al. High-resolution noise substitution to measure overfitting and validate resolution in 3D structure determination by single particle electron cryomicroscopy. *Ultramicroscopy* **135**, 24–35 (2013). 23872039 10.1016/j.ultramic.2013.06.004 PMC3834153

[69] Abramson, J. et al. Accurate structure prediction of biomolecular interactions with AlphaFold 3. *Nature* **630**, 493–500 (2024). 38718835 10.1038/s41586-024-07487-w PMC11168924

[70] Pettersen, E. F. et al. UCSF Chimera-a visualization system for exploratory research and analysis. *J. Comput. Chem.* **25**, 1605–1612 (2004). 15264254 10.1002/jcc.20084

[71] Emsley, P., Lohkamp, B., Scott, W. G. & Cowtan, K. Features and development of Coot. *Acta Crystallogr. Sect. D. Biol. Crystallogr.* **66**, 486–501 (2010). 20383002 10.1107/S0907444910007493 PMC2852313

[72] Adams, P. D. et al. PHENIX: a comprehensive Python-based system for macromolecular structure solution. *Acta Crystallogr. Sect. D. Biol. Crystallogr.* **66**, 213–221 (2010). 20124702 10.1107/S0907444909052925 PMC2815670

[73] Afonine, P. V. et al. Real-space refinement in PHENIX for cryo-EM and crystallography. *Acta Crystallogr. Sect. D. Struct. Biol.* **74**, 531–544 (2018). 29872004 10.1107/S2059798318006551 PMC6096492

[74] Goddard, T. D. et al. UCSF ChimeraX: meeting modern challenges in visualization and analysis. *Protein Sci. Publ. Protein Soc.* **27**, 14–25 (2018). 10.1002/pro.3235 PMC5734306 28710774

[75] Langley, C. A., Lilly, M., Malik, H. S. & Emerman, M. Strain-Specific Epistasis Shapes Fitness Landscapes of APOBEC3G Antagonism By HIV-1 Vif Proteins. *bioRxiv*10.1101/2025.10.20.683452 (2025). 10.1126/sciadv.aed4872 PMC13394466 42490454

[76] Vermeire, J. et al. Quantification of reverse transcriptase activity by real-time PCR as a fast and accurate method for titration of HIV, lenti- and retroviral vectors. *PLoS ONE* **7**, ARTN e50859 (2012). 10.1371/journal.pone.0050859 PMC3515444 23227216

