# Supplementary material for: The structural mechanism of HIV-2 Vif antagonism of human APOBEC3H
Source: Nat Commun. 2026 Jun 29;17:8079. doi: 10.1038/s41467-026-75045-1 (PMC13454587; doi:10.1038/s41467-026-75045-1)

Supplementary Information

The structural mechanism of HIV-2 Vif antagonism of human APOBEC3H

Yange Niu[1], Michelle Lilly[2], Estelle K. Ronayne[1,3], Michael Emerman[2], Nicholas M. Chesarino[4], John D. Gross[1,5]*

[1] Department of Pharmaceutical Chemistry, University of California, San Francisco, San Francisco, CA, USA

[2] Divisions of Human Biology and Basic Sciences, Fred Hutchinson Cancer Center, Seattle, WA, USA

[3] Biophysics graduate program, University of California, San Francisco, San Francisco, CA, USA

[4] Department of Biology, University of Akron, Akron, OH, USA

[5] Quantitative Bioscience Institute, University of California, San Francisco, San Francisco, CA, USA

* Corresponding author: jdgross@cgl.ucsf.edu (J.D.G.)

Table of contents:

Supplementary Figs.1-10

Supplementary Tables 1-2

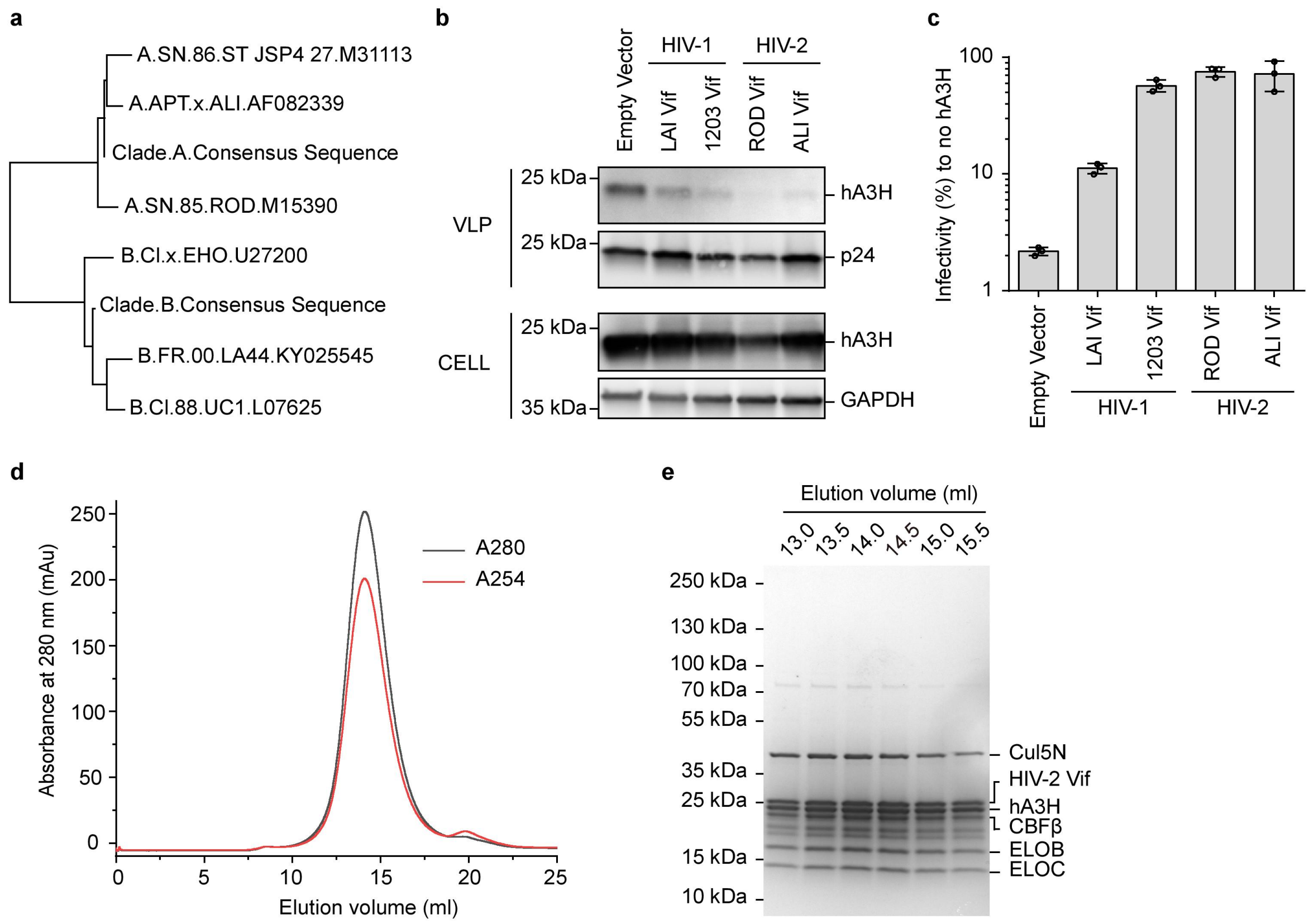

Supplementary Fig.1: Biochemical characterization of HIV-2 and HIV-1 Vif, and purification of the hA3H-VCBC complex. **(a)** The phylogenetic tree of selected HIV-2 Vif sequences. Consensus sequences for clade A and clade B were generated from all available HIV-2 Vif sequences in the Los Alamos National Laboratory HIV Sequence Database. **(b)** Immunoblot analysis of hA3H incorporation into VLPs in the presence of HIV-1 Vif from lab (LAI) and adapted (1203) strains, and HIV-2 Vif from lab (ROD) and primary isolate (ALI) strains. The experiment was repeated twice independently with similar results. **(c)** Effects of HIV-1 and HIV-2 Vif (lab and adapted strains) on HIV-1 ΔVif infectivity in the presence of hA3H. The Y-axis is the % of the RLUs of the viral infections from virus generated in the presence of A3H relative to viral infections from virus generated in the absence of A3H. The viruses were normalized to equal reverse transcriptase activity prior to infection. Data are shown as means ± standard deviations; n = 3 biologically independent samples. **(d)** Size-exclusion chromatography profile of the purified hA3H-VCBC complex. Absorbance at A_{280} and A_{254} is shown in black and red, respectively. **(e)** SDS-PAGE analysis of the purified hA3H-VCBC complex. Source data are provided at the end of the Supplementary Information file.

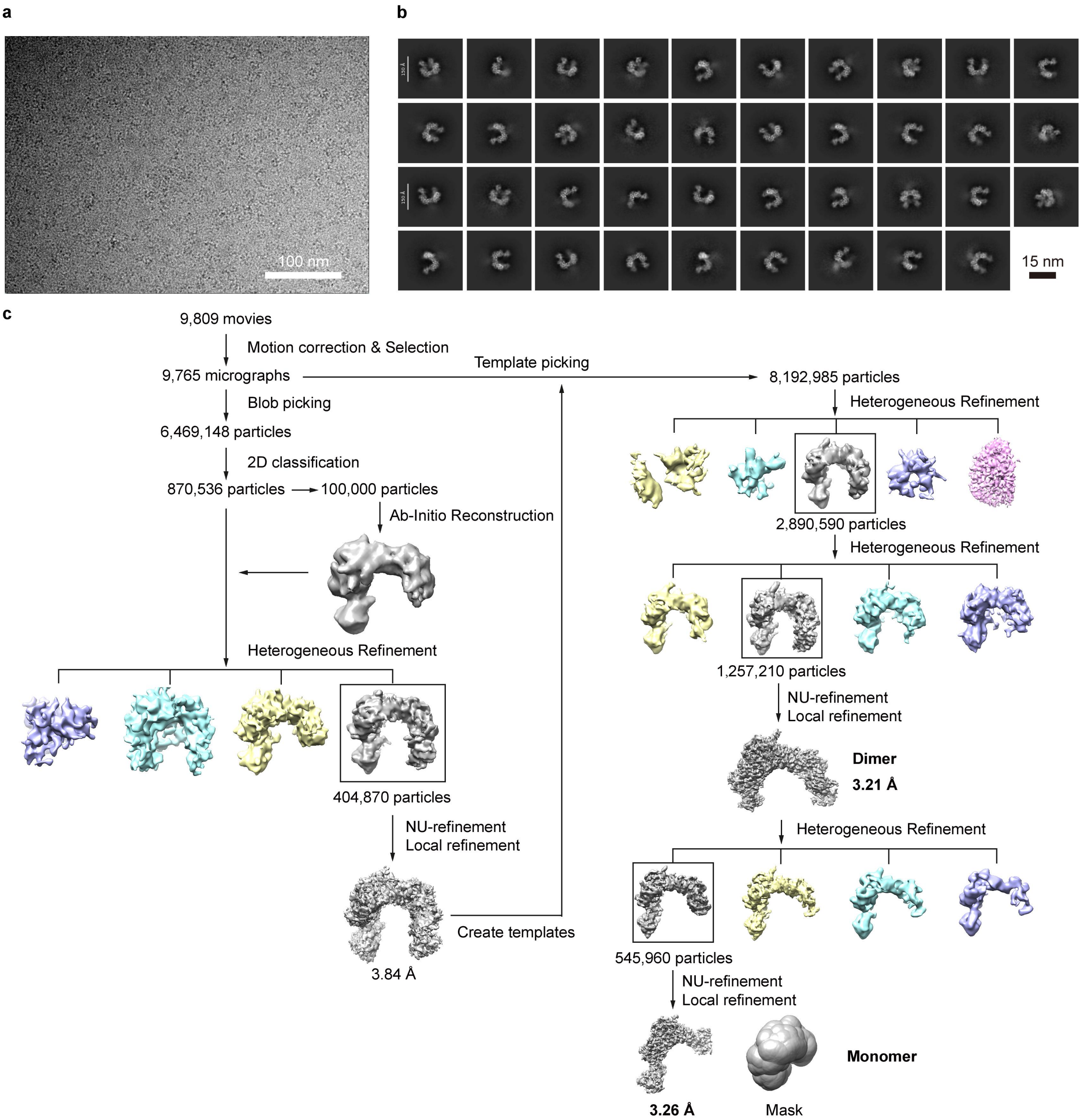

Supplementary Fig.2: Cryo-EM data processing of the hA3H-VCBC complex.

(a) Representative cryo-EM micrograph. **(b)** 2D class averages generated in cryoSPARC. **(c)** Data processing workflow for reconstruction of the hA3H-VCBC complex.

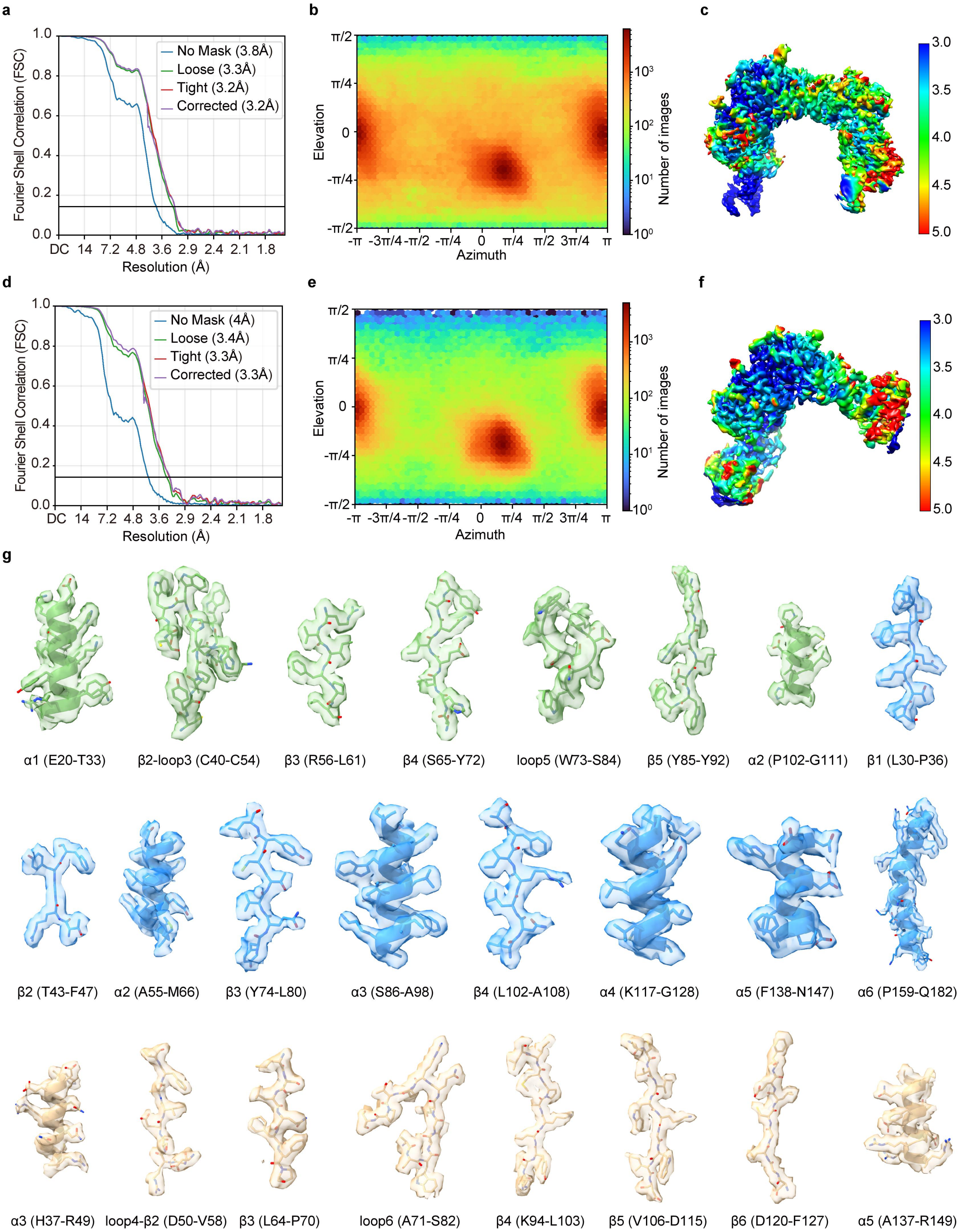

a
Fourier Shell Correlation (FSC)
No Mask (3.8Å)
Loose (3.3Å)
Tight (3.2Å)
Corrected (3.2Å)
Resolution (Å)
DC 14 7.2 4.8 3.6 2.9 2.4 2.1 1.8
b
Elevation
Azimuth
Number of images
c
3.0 3.5 4.0 4.5 5.0
d
No Mask (4Å)
Loose (3.4Å)
Tight (3.3Å)
Corrected (3.3Å)
e
f
g
α1 (E20-T33)
β2-loop3 (C40-C54)
β3 (R56-L61)
β4 (S65-Y72)
loop5 (W73-S84)
β5 (Y85-Y92)
α2 (P102-G111)
β1 (L30-P36)
β2 (T43-F47)
α2 (A55-M66)
β3 (Y74-L80)
α3 (S86-A98)
β4 (L102-A108)
α4 (K117-G128)
α5 (F138-N147)
α6 (P159-Q182)
α3 (H37-R49)
loop4-β2 (D50-V58)
β3 (L64-P70)
loop6 (A71-S82)
β4 (K94-L103)
β5 (V106-D115)
β6 (D120-F127)
α5 (A137-R149)

Supplementary Fig.3: Cryo-EM density and model validation of the HIV-2 Vif-hA3H-CBFβ complex.

(a) Gold-standard FSC curve for the dimeric complex (0.143 cutoff). **(b)** Angular distribution of particles for the dimeric complex. **(c)** Local resolution map of the dimeric complex calculated in cryoSPARC. **(d-f)** As in **(a-c)** but for the monomeric complex. **(g)** Cryo-EM density and refined atomic model of the monomeric HIV-2 Vif-hA3H-CBFβ complex. HIV-2 Vif, CBFβ, and hA3H are colored as in Fig.1.

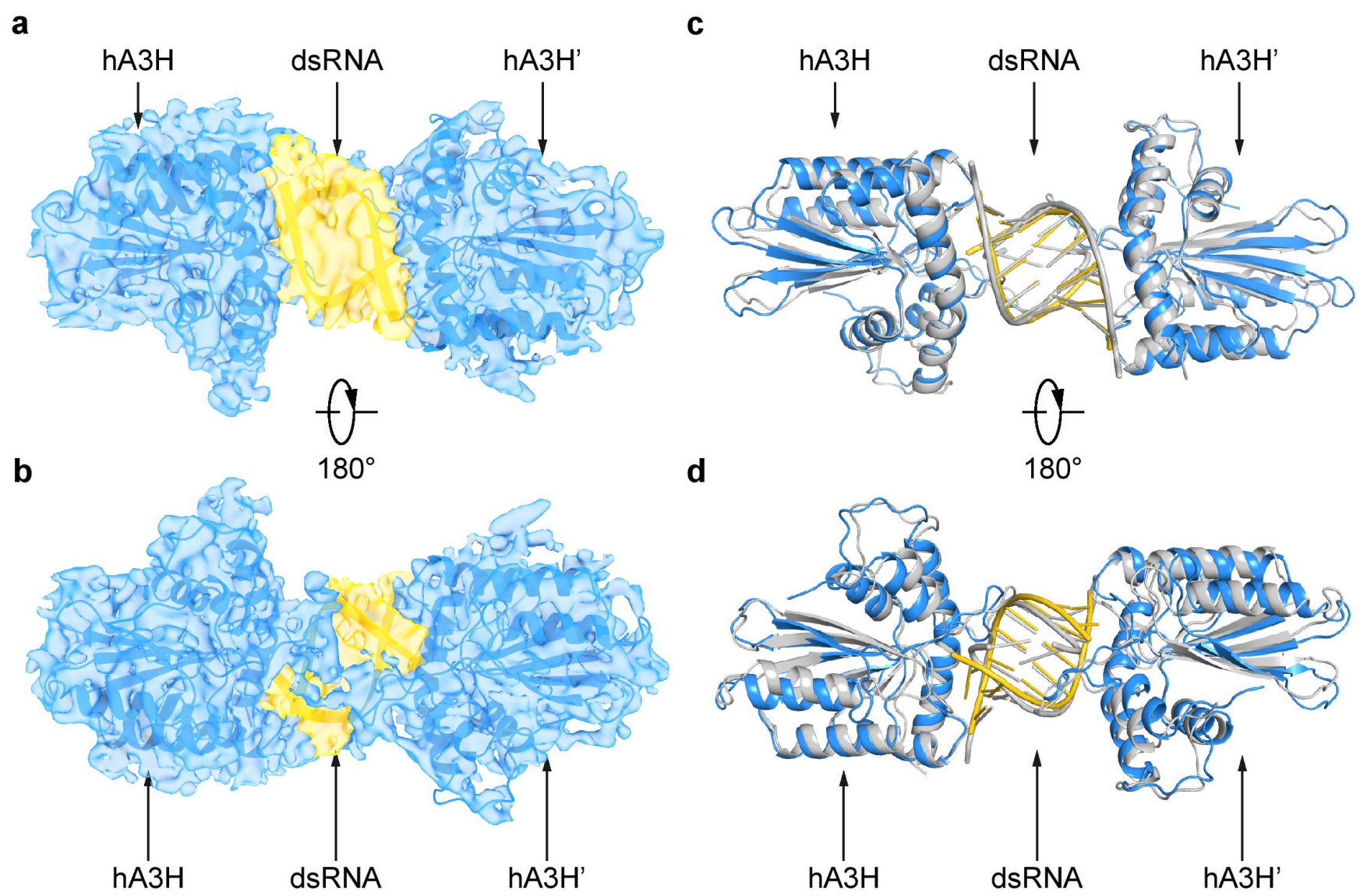

Supplementary Fig.4: Structure of the hA3H dimer.

(a) Cryo-EM density and model of the hA3H dimer bound to dsRNA. **(b)** 180° rotated view of **(a)**. **(c)** Structural comparison of hA3H dimer in the HIV-2 Vif complex (colored) with the hA3H dimer crystal structure (grey; PDB: 5W3V). **(d)** 180° rotated view of **(c)**.

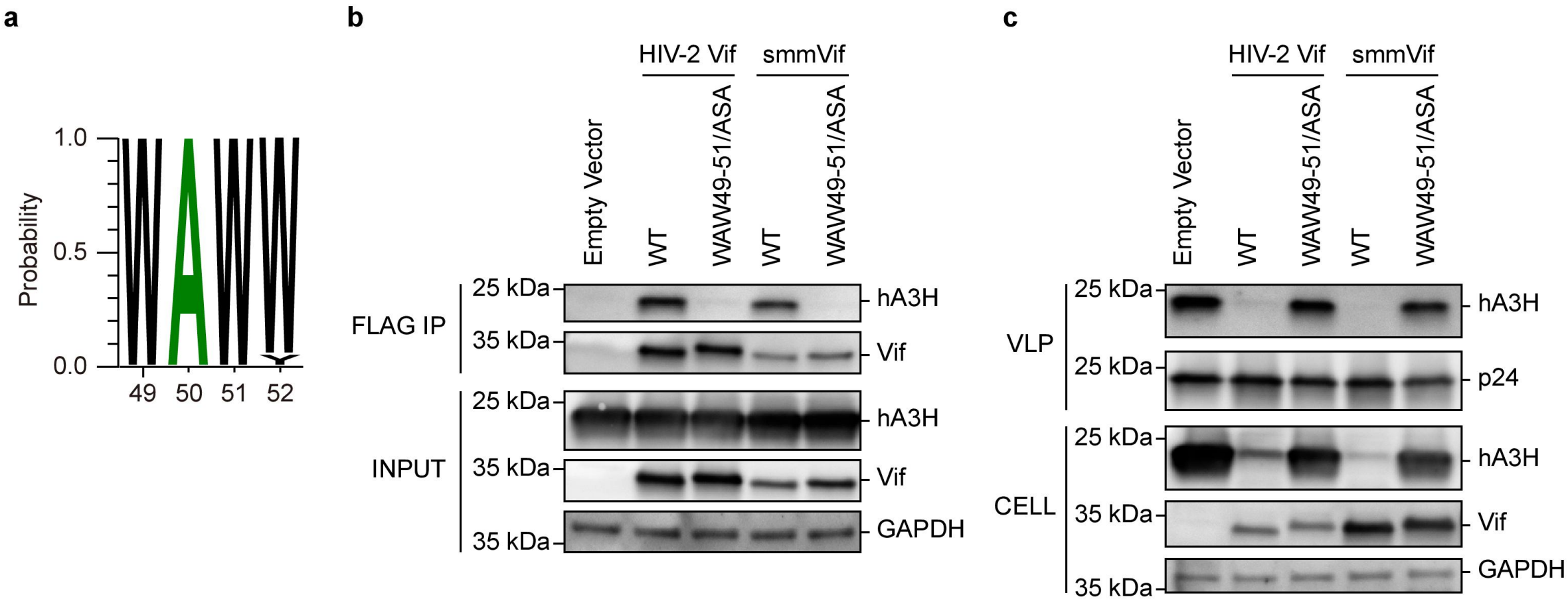

Supplementary Fig.5: Biochemical comparison of HIV-2 Vif and smmVif.

(a) Conservation of W-rich motif in smmVif. A total of 19 smmVif sequences were retrieved from the Los Alamos National Laboratory HIV Sequence Database and aligned using MEGA-X. Sequence conservation was visualized as a sequence logo generated with the WebLogo 3 server. **(b)** Co-immunoprecipitation of hA3H with HIV-2 Vif and smmVif containing the WAW49-51/ASA mutation. **(c)** Immunoblot analysis of hA3H incorporation into VLPs in the presence of HIV-2 Vif or smmVif WAW49-51/ASA mutants. Supplementary Fig.5b-c were repeated twice independently with similar results. Source data are provided at the end of the Supplementary Information file.

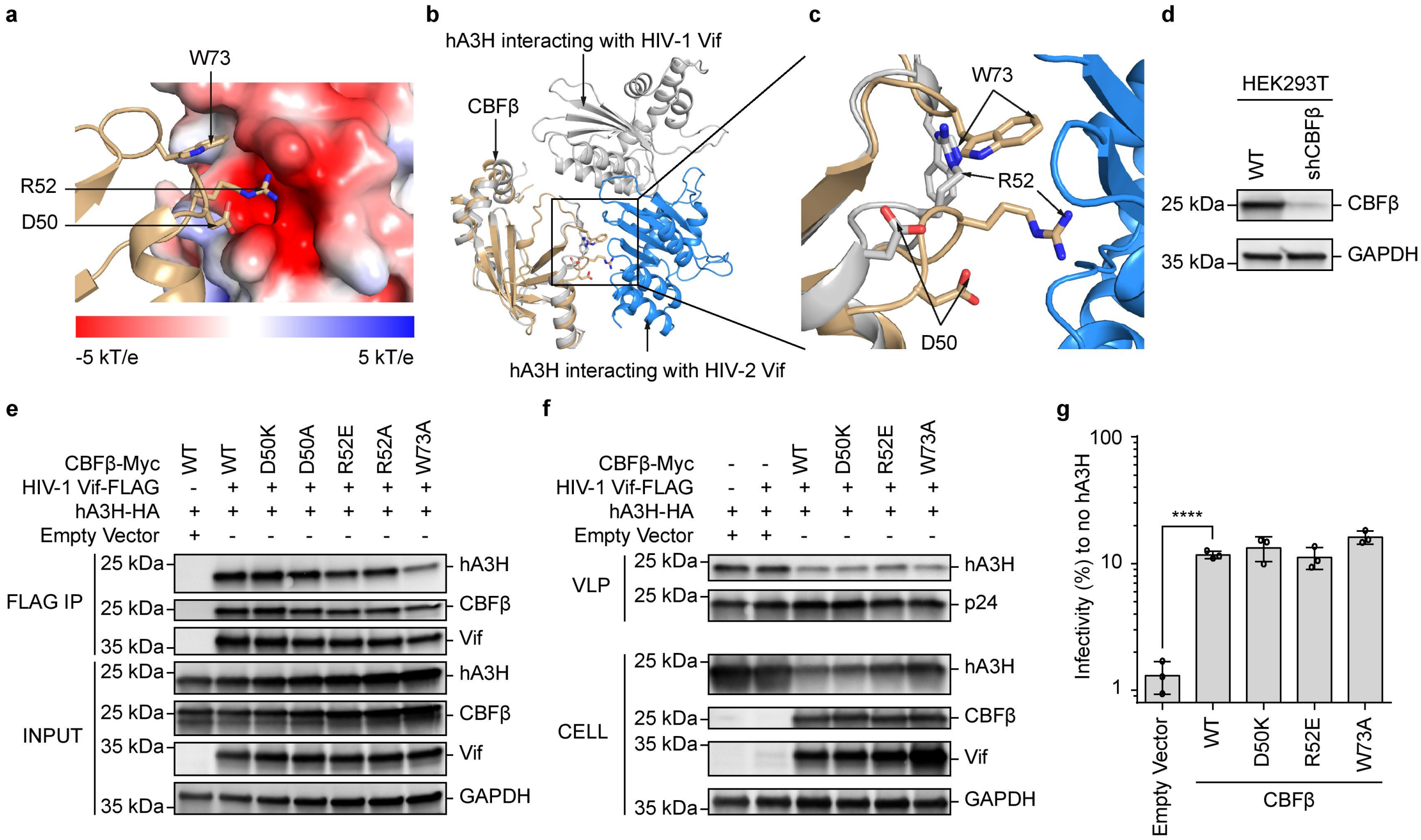

Supplementary Fig.6: CBFβ-hA3H interaction in the HIV-2 Vif complex.

(a) CBFβ-hA3H binding interface. hA3H surface is colored by electrostatic potential, and interacting CBFβ residues are shown as sticks. **(b-c)** Structural comparison of the CBFβ-hA3H interface in HIV-2 Vif complex (colored) and HIV-1 Vif complex (grey; PDB: 8FVI) complexes, with a close-up view in **(c)**. **(d)** Immunoblot of CBFβ expression in HEK293T WT and shCBFβ cells. **(e)** Co-immunoprecipitation of CBFβ mutants and hA3H in the presence of HIV-1 (LAI) Vif. **(f)** Immunoblot analysis of hA3H incorporation into VLPs in the presence of HIV-1 (LAI) Vif and CBFβ mutants. **(g)** Effect of CBFβ mutants on HIV-1 ΔVif infectivity in the presence of HIV-1 (LAI) Vif and hA3H. Viruses were produced in 293T cells knocked down for endogenous CBFβ and then complemented with empty vector, CBFβ WT, or CBFβ mutants. The Y-axis is the % of the RLUs of the viral infections from virus generated in the presence of A3H relative to viral infections from virus generated in the absence of A3H. The viruses were normalized to equal reverse transcriptase activity prior to infection. Data are shown as means ± standard deviations; n = 3 biologically independent samples. Significance was determined using unpaired two-tailed Student's t-tests (****p < 0.0001). Supplementary Fig.6d-f were repeated twice independently with similar results. Source data are provided at the end of the Supplementary Information file.

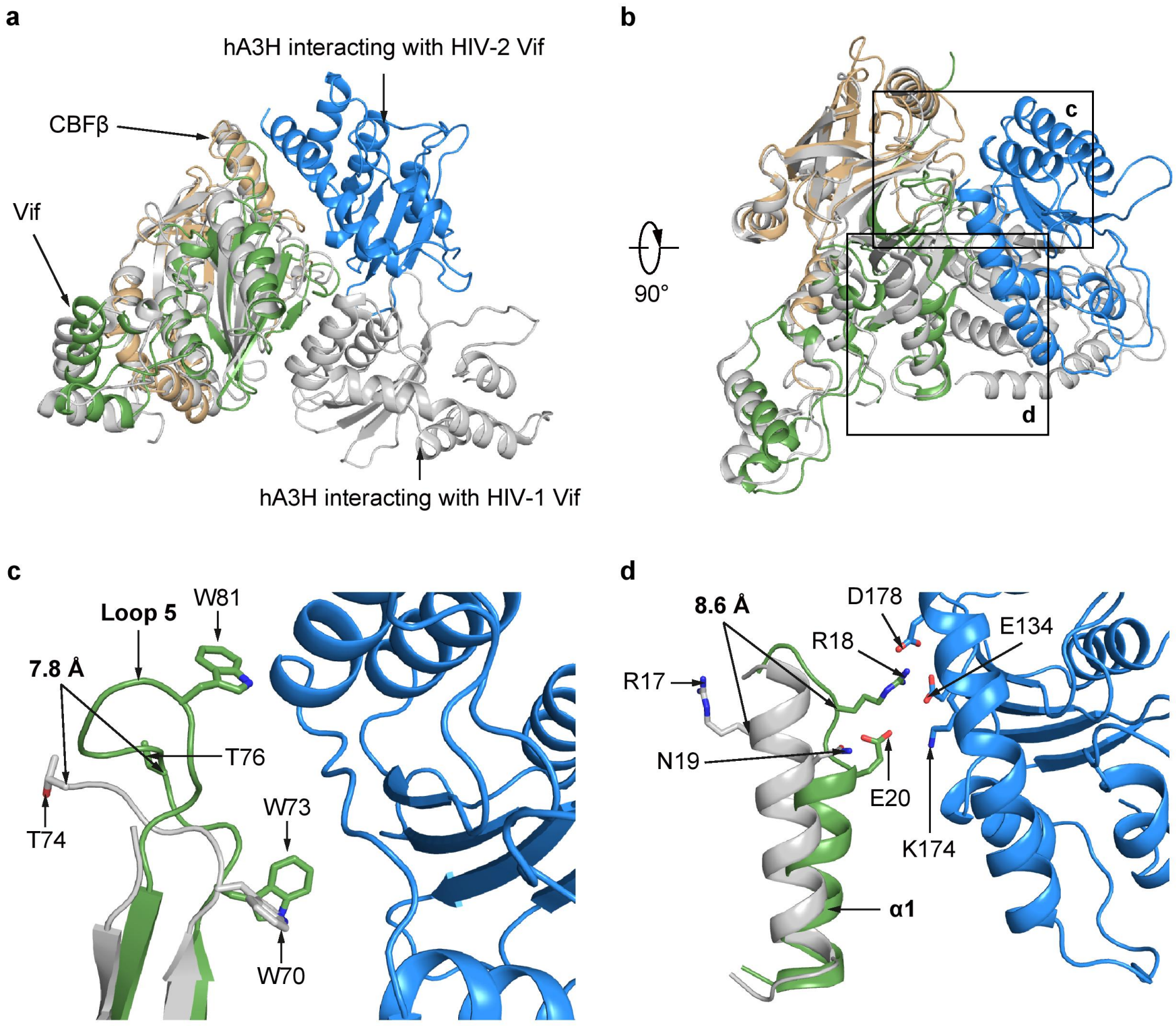

Supplementary Fig.7: Structural comparison of hA3H in complex with HIV-2 versus HIV-1 Vif.

(a) Superposition of HIV-2 Vif-hA3H (colored) and HIV-1 Vif-hA3H (grey) complexes aligned on Vif-CBFβ. **(b)** 90° rotated view of **(a)**. **(c)** Close-up view of Vif loop 5 conformational differences. **(d)** Close-up view of Vif helix α1 conformational differences.

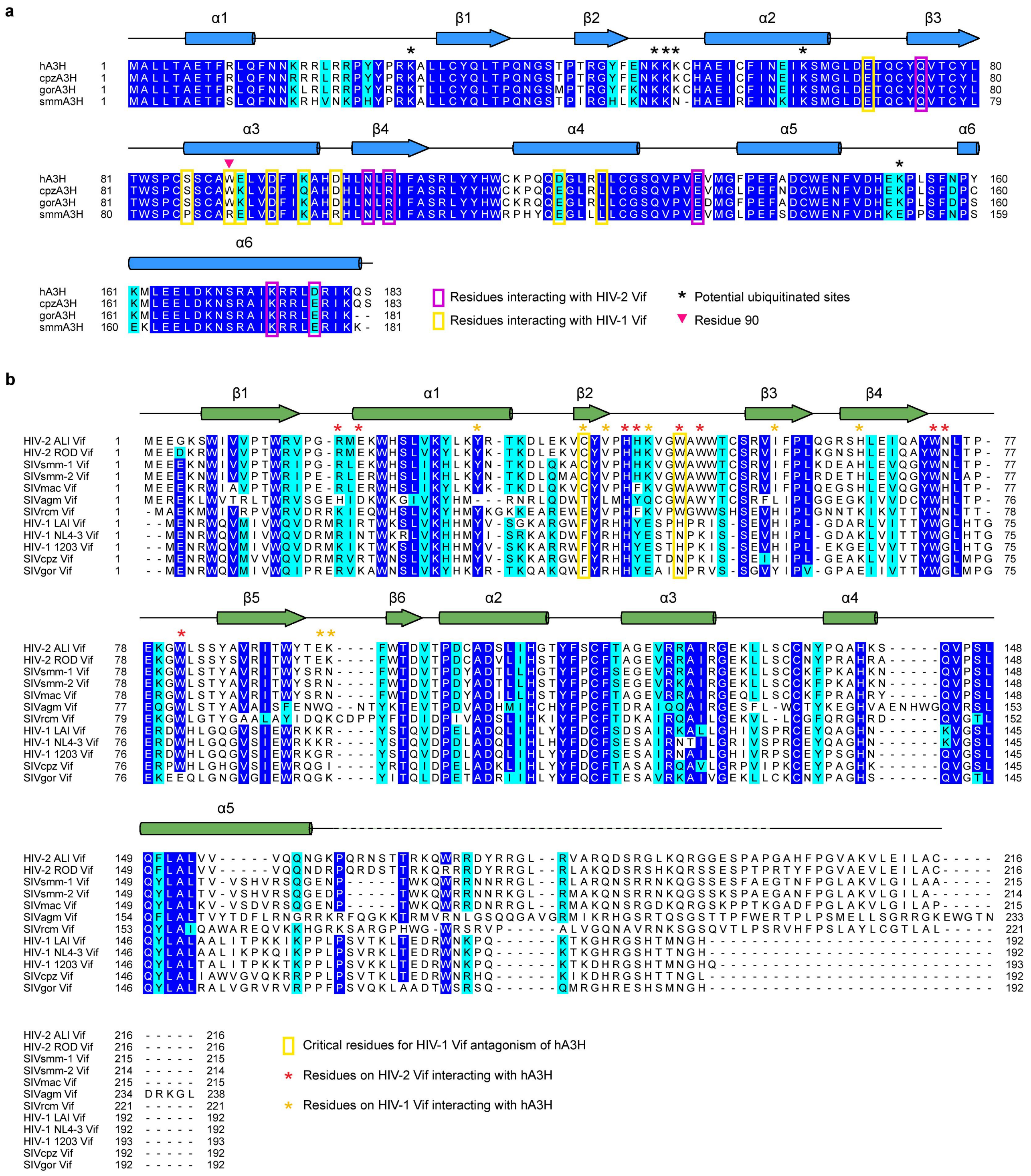

Supplementary Fig.8: Sequence alignments of A3H and Vif.

(a) Alignment of A3H from human, chimpanzee, gorilla and sooty mangabey. Helices and β-strands are indicated as cylinders and arrows, respectively. Conservation is shaded from cyan to blue. Residues contacting HIV-2 Vif are boxed in red; residues contacting HIV-1 Vif are boxed in yellow. Potential ubiquitinated sites on A3H are indicated as asterisks. The W90 residue of hA3H is marked with a red triangle. **(b)** Alignment of Vif sequences from HIV-2, HIV-1, and SIV by MEGA-X. Helices and β-strands are indicated as cylinders and arrows, respectively; unmodeled regions as dashed lines. Conservation is shaded from cyan to blue. HIV-2 Vif residues contacting hA3H are marked with red asterisks; HIV-1 Vif residues contacting hA3H with yellow asterisks. Residues critical for HIV-1 Vif antagonism of A3H are boxed in yellow.

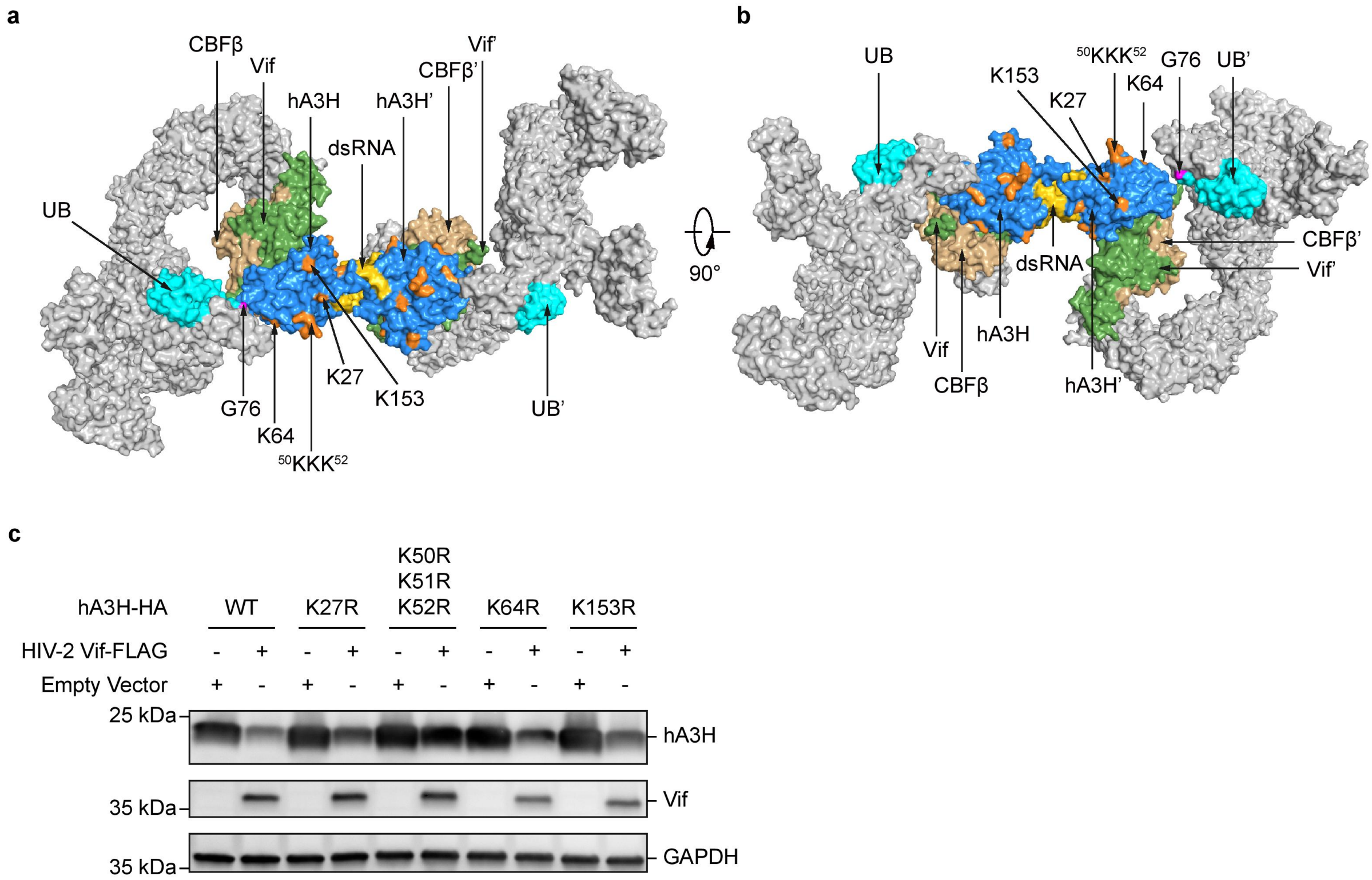

Supplementary Fig.9: Proposed model of hA3H ubiquitination.

(a) Dimeric HIV-2 Vif-hA3H-CBFβ complex overlaid with VCBC-CUL5$_{NTD}$ (PDB: 4N9F), neddylated CUL5$_{CTD}$-RBX2-ARIH2 (PDB: 7ONI), and a comparative ARIH2-Ub model based on CUL1-RBX1-ARIH1-Ub (PDB: 7B5M). HIV-2 Vif, CBFβ, hA3H, and dsRNA are colored as in Fig.1. Ubiquitin is shown as cyan with the terminal glycine in magenta. Surface-exposed lysine residues on hA3H are highlighted in orange. **(b)** A 90° rotated view of **(a)**. **(c)** HIV-2 Vif-mediated degradation of wild-type hA3H and lysine-to-arginine mutants. The experiment was repeated twice independently with similar results. Source data are provided at the end of the Supplementary Information file.

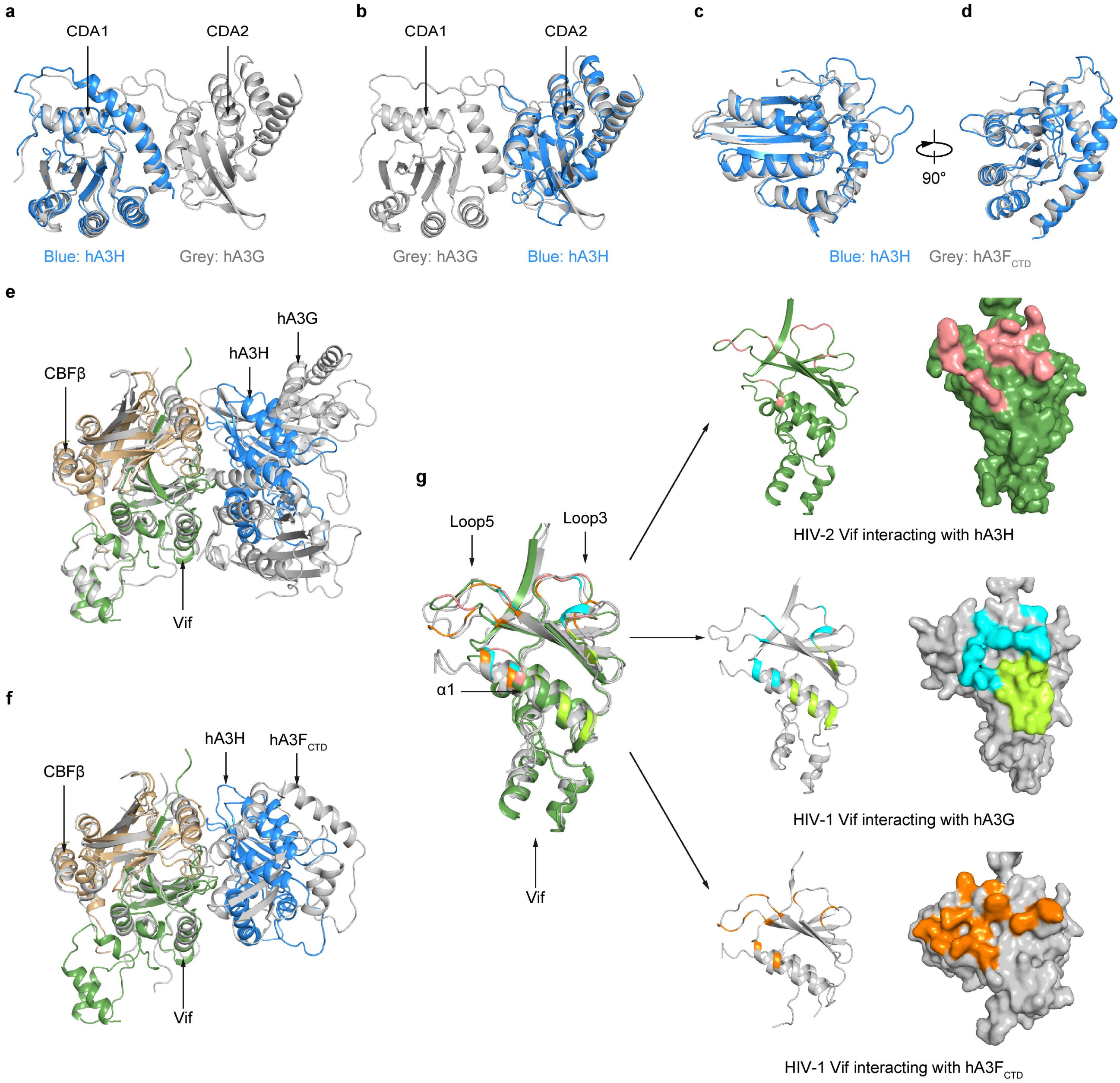

Supplementary Fig.10: Comparison of HIV-2 Vif-hA3H complex with HIV-1 Vif-hA3G and HIV-1 Vif-hA3F$_{CTD}$ complexes.
(a-b) Superposition of hA3H (blue) with hA3G CDA1 (grey, **a**) or CDA2 (grey, **b**) from the HIV-1 Vif-hA3G complex (PDB: 8CX0). **(c)** Superposition of hA3H (blue) with hA3F$_{CTD}$ (grey) from the HIV-1 Vif-hA3F$_{CTD}$ complex (PDB: 6NIL). **(d)** A 90° rotated view of **(c)**. **(e-f)** Superposition of the HIV-2 Vif-hA3H complex (colored) with HIV-1 Vif-hA3G (PDB: 8CX0, **e**) or HIV-1 Vif-hA3F$_{CTD}$ (PDB: 6NIL, **f**) complexes aligned on Vif-CBFβ. **(g)** Structural alignment of Vif across the three complexes. HIV-2 Vif residues contacting hA3H are colored as salmon; HIV-1 Vif residues contacting hA3G are colored as cyan; residues contacting RNA are colored as lime; and residues contacting hA3F$_{CTD}$ are colored as orange.

Supplementary Table 1. Cryo-EM data collection, refinement and validation statistics

	HIV-2 Vif-A3H-CBFβ monomer	HIV-2 Vif-A3H-CBFβ dimer
**Data collection and processing**		
Magnification	105,000×	
Voltage (kV)	300	
Electron exposure (e–/Å^2)	47.7	
Defocus range (μm)	-1.2 to -2.5	
Pixel size (Å)	0.8189	
Symmetry imposed	*C1*	
Initial particle images (no.)	8,192,985	
Final particle images (no.)	545,960	1,257,210
Map resolution (Å)	3.26	3.21
FSC threshold	0.143	0.143
Map resolution range (Å)	200-3.26	200-3.21
**Refinement**		
Initial model used (PDB code)		
Model resolution (Å)	3.2	3.2
FSC threshold	0.143	0.143
Model resolution range (Å)	200-3.2	200-3.2
Map sharpening *B* factor (Å^2)	-95.5	-118.8
Model composition		
Non-hydrogen atoms	4,557	8,775
Protein residues	509	1018
Nucleotides	16	16
Ligands	2 Zn	2 Zn
B factors (Å^2)		
Protein	54.55	54.55
Nucleotides	76.58	76.58
Ligand	109.06	109.06
R.m.s. deviations		
Bond lengths (Å)	0.004	0.004
Bond angles (°)	0.935	0.954
Validation		
MolProbity score	1.55	1.67
Clashscore	5.42	7.44
Poor rotamers (%)	0.22	0.22
Ramachandran plot		
Favored (%)	96.21	96.21
Allowed (%)	3.79	3.79
Disallowed (%)	0	0

Supplementary Table 2. Oligonucleotides used in this study

Oligonucleotide Name	Oligonucleotide DNA sequence (5' ->3')
HIV-2 Vif R18A/E20A-Fwd	TTGGAGGGTGCCAGGGGCGATGGCGAAGTGGCATAGCCTTGTCAAGTA
HIV-2 Vif R18A/E20A-Rev	AAGGCTATGCCACTTCGCCATCGCCCCTGGCACCCTCCAAGTGGGGA
HIV-2 Vif H44A/H45A-Fwd	GGTGTGCTATGTTCCCGCCGCTAAGGTGGGATGGGCATGGTGGACT
HIV-2 Vif H44A/H45A-Rev	TGCCCATCCCACCTTAGCGGCGGGAACATAGCACACCTTTTCTAGG
HIV-2 Vif WAW49-51/ASA-Fwd	CCACCATAAGGTGGGAGCGTCAGCGTGGACTTGCAGCAGGGTAATATTC
HIV-2 Vif WAW49-51/ASA-Rev	CCTGCTGCAAGTCCACGCTGACGCTCCCACCTTATGGTGGGGAACATAG
HIV-2 Vif W73A/N74A-Fwd	AGAGATACAGGCATATGCGGCCCTAACACCAGAAAAAGGA
HIV-2 Vif W73A/N74A-Rev	CTTTTTCTGGTGTTAGGGCCGCATATGCCTGTATCTCTAGGTGACTT
HIV-2 Vif W81A/L82A-Fwd	AACACCAGAAAAAGGAGCGGCCTCCTCTTATGCAGTAAGAATAACTT
HIV-2 Vif W81A/L82A-Rev	TACTGCATAAGAGGAGGCCGCTCCTTTTTCTGGTGTTAG
CBFβ D50A-Fwd	CAGAACGCCTGCCGCGCCGGCCGCTCGGAAATCGCTT
CBFβ D50A-Rev	GATTTCCGAGCGGCCGGCGCGGCAGGCGTTCTGGAAG
CBFβ D50K-Fwd	CCAGAACGCCTGCCGCAAGGGCCGCTCGGAAATCGCTTT
CBFβ D50K-Rev	CGATTTCCGAGCGGCCCTTGCGGCAGGCGTTCTGGAAGC
CBFβ R52E-Fwd	CGCCTGCCGCGACGGCGAGTCGGAAATCGCTTTTGTGGC
CBFβ R52E-Rev	CAAAAGCGATTTCCGACTCGCCGTCGCGGCAGGCGTTCT
CBFβ R52A-Fwd	CGCCTGCCGCGACGGCGCCTCGGAAATCGCTTTTGTGG
CBFβ R52A-Rev	AAAAGCGATTTCCGAGGCGCCGTCGCGGCAGGCGTTCT
CBFβ W73A-Fwd	GTTTTTTCCGGCCAGCGCGCAGGGAGAACAGCGACAAA
CBFβ W73A-Rev	TCGCTGTTCTCCCTGCGCGCTGGCCGGAAAAAACTGGA
hA3H W90A-Fwd	CTGCTCCTCCTGTGCCGCGGAGCTGGTTGACTTCATC
hA3H W90A-Rev	GAAGTCAACCAGCTCCGCGGCACAGGAGGAGCAGGGGC
smmVif WAW49-51/ASA-Fwd	GCATCACAAGGTCGGGGCGTCAGCGTGGACCTGTAGTCGGGTGATATTT
smmVif WAW49-51/ASA-Rev	CCGACTACAGGTCCACGCTGACGCCCCGACCTTGTGATGCGGTACATAA
hA3H K27R-Fwd	CCTTACTACCCGAGGAGGGCCCTCTTGTGTTACCAGC
hA3H K27R-Rev	GTAACACAAGAGGGCCCTCCTCGGGTAGTAAGGCCTT
hA3H K50R/K51R/K52R-Fwd	GGCTACTTTGAAAACAGGAGAAGGTGCCATGCGGAAATTTGCTTTATT
hA3H K50R/K51R/K52R-Rev	AATTTCCGCATGGCACCTTCTCCTGTTTTCAAAGTAGCCTCTCGTGGG
hA3H K64R-Fwd	TTTATTAACGAGATCAGGTCCATGGGACTGGACGAAA
hA3H K64R-Rev	GTCCAGTCCCATGGACCTGATCTCGTTAATAAAGCA
hA3H K153R-Fwd	TTTGTGGACCACGAGAGACCGCTTTCCTTCAACCCCT
hA3H K153R-Rev	GTTGAAGGAAAGCGGTCTCTCGTGGTCCACAAAGTTT

Source Data for Supplementary Information

Supplementary Fig.1

Supplementary Fig.1b

VLP:
anti-HA (Cell Signaling Technology, 3724)

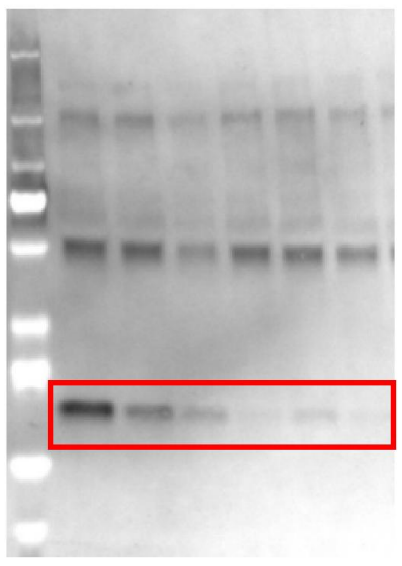

VLP:
anti-p24gag (BEI Resources, 3537)

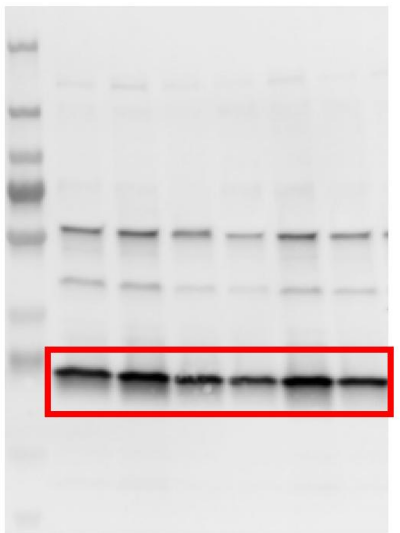

CELL:
anti-HA (Cell Signaling Technology, 3724)

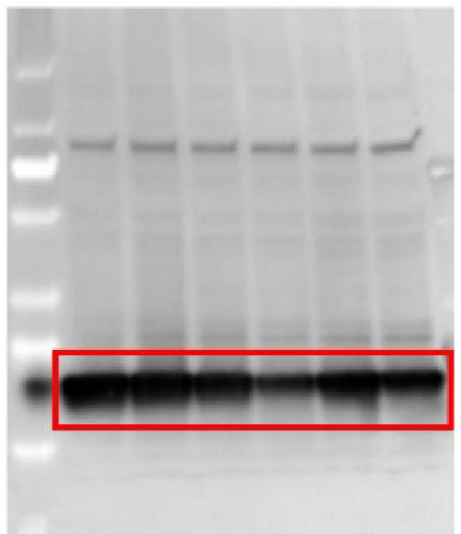

CELL:
anti-GAPDH (Proteintech Group, 60004-1-Ig

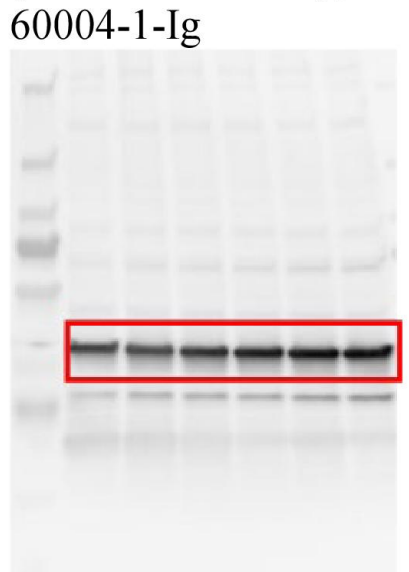

Supplementary Fig.1c

		Rep 1	Rep 2	Rep 3
	Empty Vector	1.983451	2.180937	2.335492
	LAI Vif	9.960174	12.19938	11.40323
HIV-1	1203 Vif	56.52006	64.29179	51.14188
	ROD Vif	79.80193	79.27414	66.92761
HIV-2	ALI Vif	92.20218	72.91102	50.83542

Supplementary Fig.1e

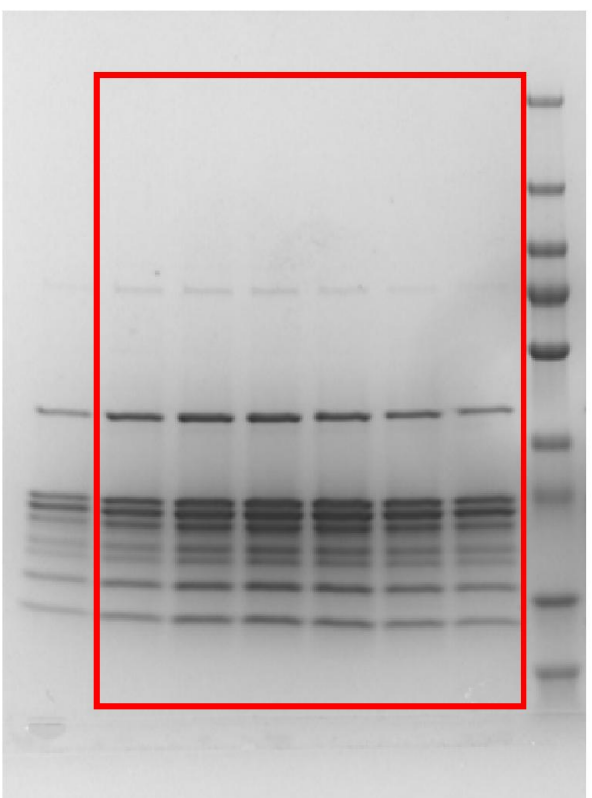

Supplementary Fig.5

Supplementary Fig.5b

FLAG IP:
anti-HA (Cell Signaling Technology, 3724)

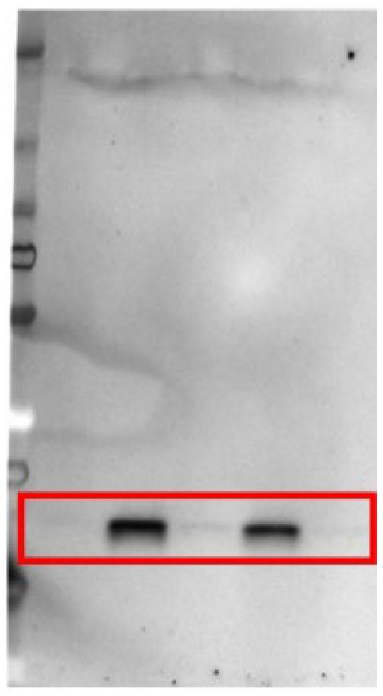

FLAG IP:
anti-FLAG (Sigma-Aldrich, F3165)

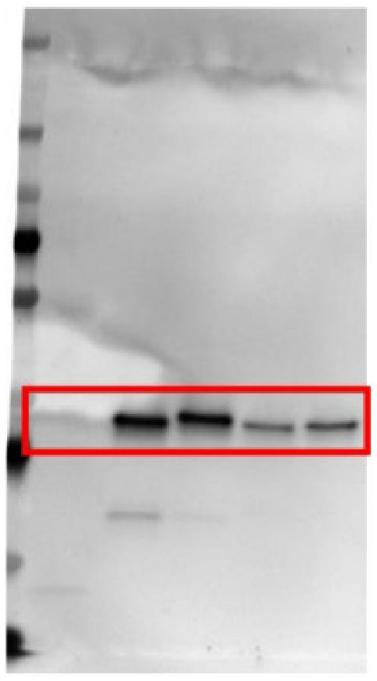

INPUT:
anti-HA (Cell Signaling Technology, 3724)

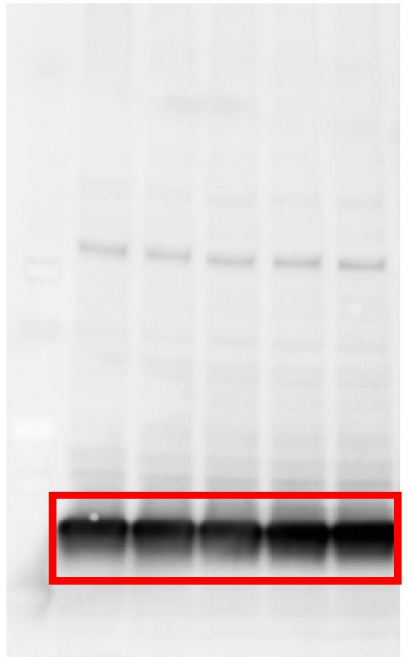

INPUT:
anti-FLAG (Sigma-Aldrich, F3165)

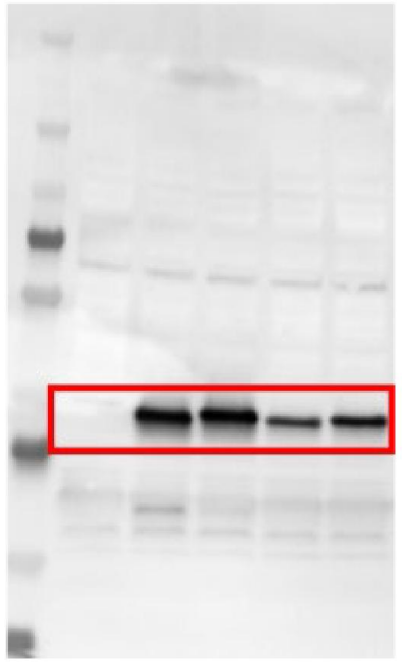

INPUT:
anti-GAPDH (Proteintech Group, 60004-1-Ig)

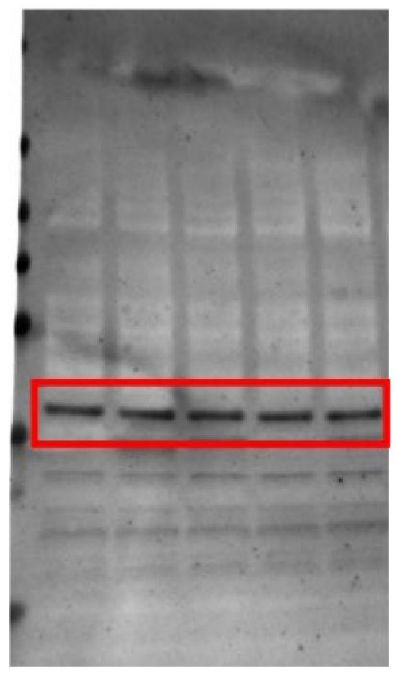

Supplementary Fig.5c

VLP:
anti-HA (Cell Signaling Technology, 3724)

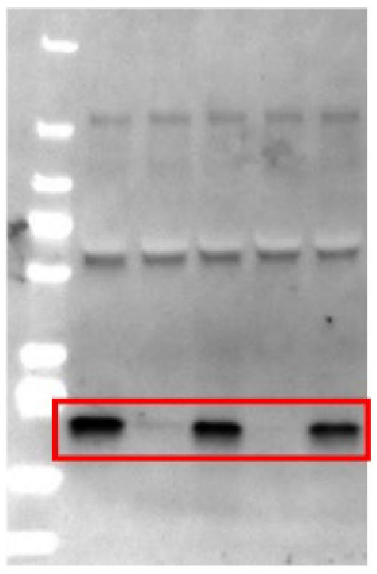

VLP:
anti-p24gag (BEI Resources, 3537)

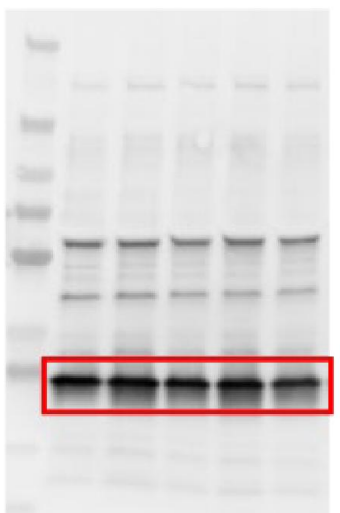

CELL:
anti-HA (Cell Signaling Technology, 3724)

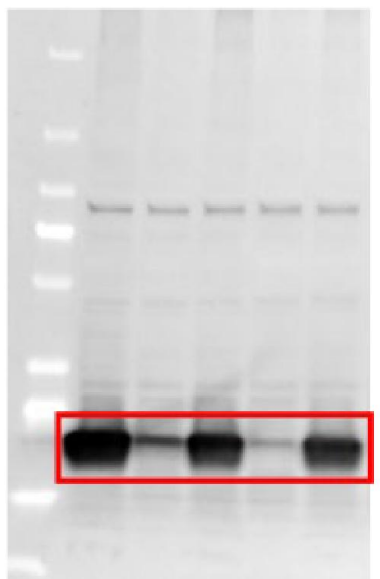

CELL:
anti-FLAG (Sigma-Aldrich, F3165)

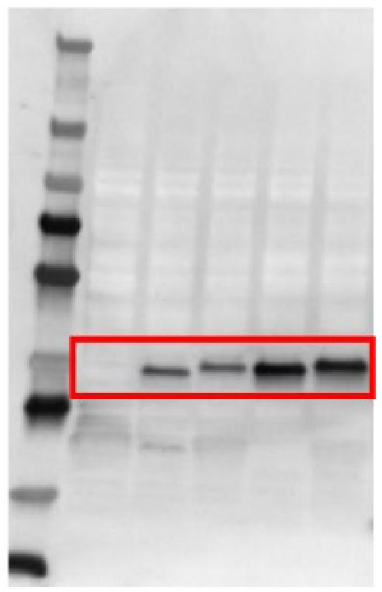

CELL:
anti-GAPDH (Proteintech Group, 60004-1-Ig)

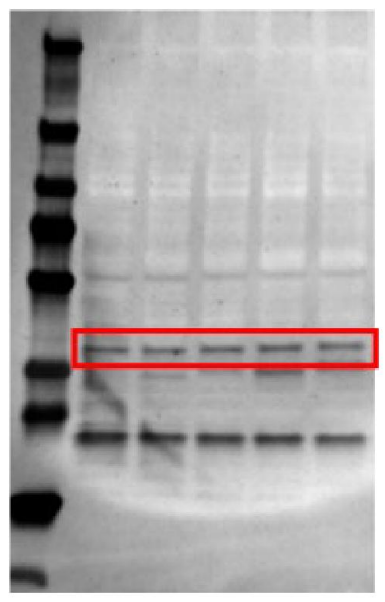

Supplementary Fig.6

Supplementary Fig.6d

CELL LYSATE:
anti-CBFβ (Santa Cruz Biotechnology, sc-166142)

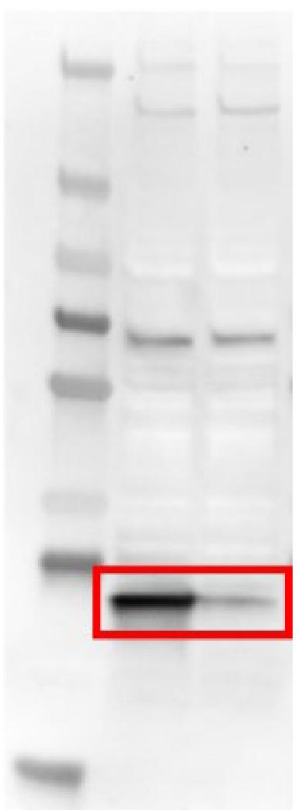

CELL LYSATE:
anti-GAPDH (Proteintech Group, 60004-1-Ig)

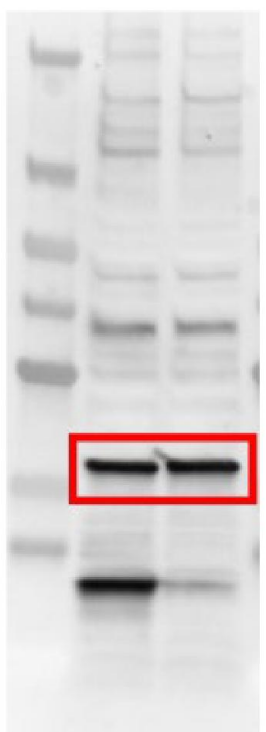

Supplementary Fig.6e

FLAG IP:
anti-HA (Cell Signaling Technology, 3724)

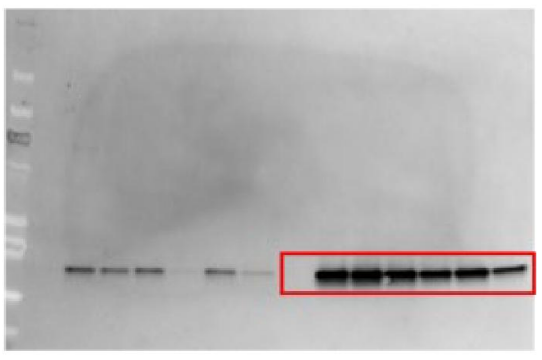

FLAG IP:
anti-CBFβ (Santa Cruz Biotechnology, sc-166142)

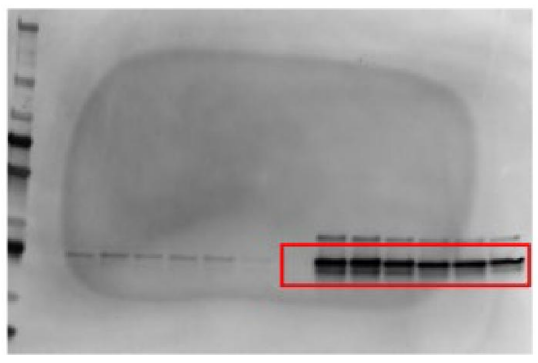

FLAG IP:
anti-FLAG (Sigma-Aldrich, F3165)

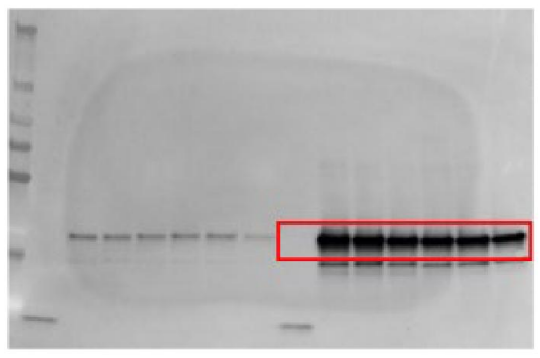

INPUT:
anti-HA (Cell Signaling Technology, 3724)

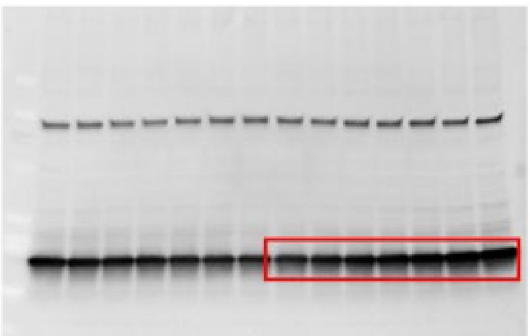

INPUT:
anti-CBFβ (Santa Cruz Biotechnology, sc-166142)

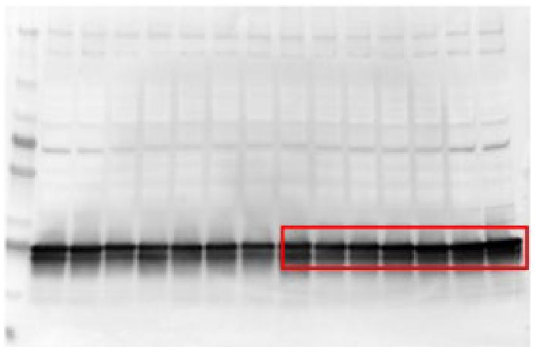

INPUT:
anti-FLAG (Sigma-Aldrich, F3165)

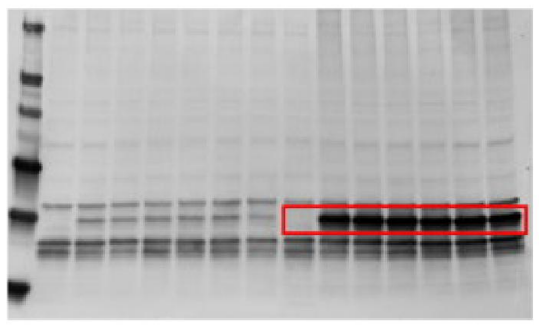

INPUT:
anti-GAPDH (Proteintech Group, 60004-1-Ig)

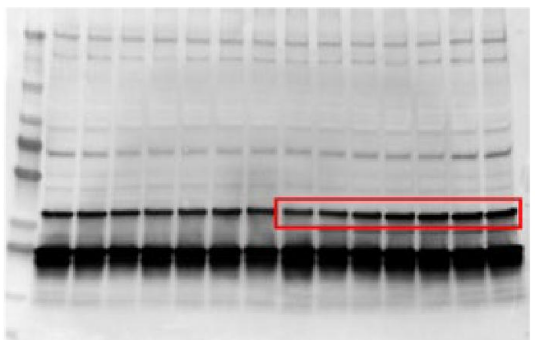

Supplementary Fig.6f

VLP:
anti-HA (Cell Signaling Technology, 3724)

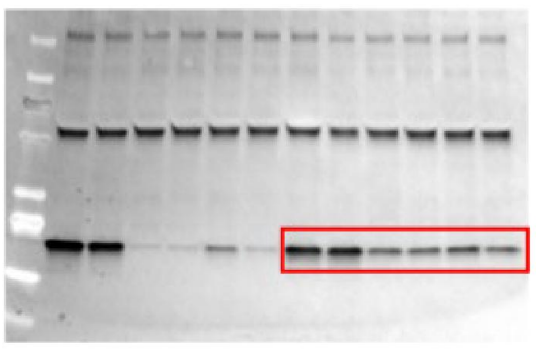

VLP:
anti-p24gag (BEI Resources, 3537)

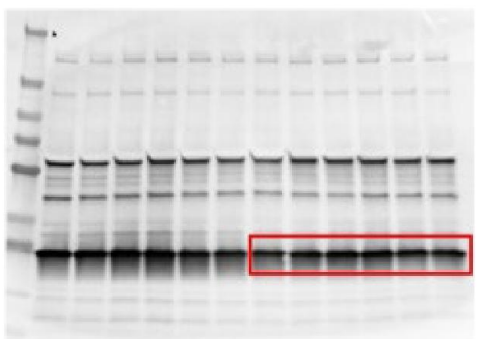

CELL:
anti-HA (Cell Signaling Technology, 3724)

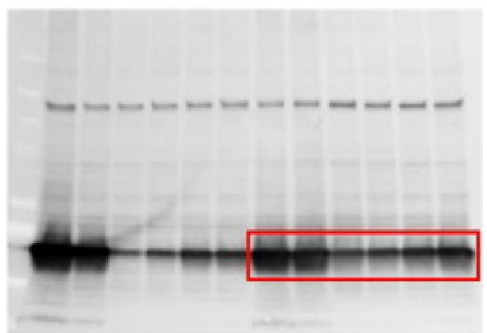

CELL:
anti-CBFβ (Santa Cruz Biotechnology, sc-166142)

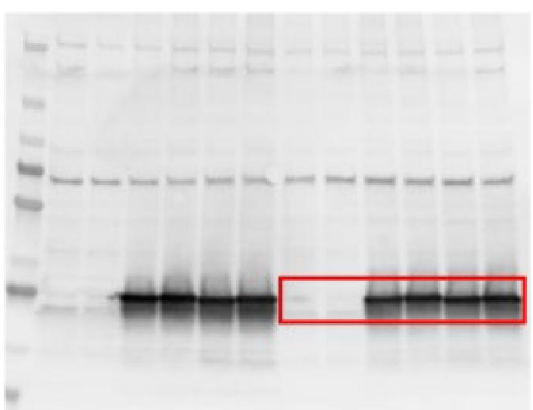

CELL:
anti-FLAG (Sigma-Aldrich, F3165)

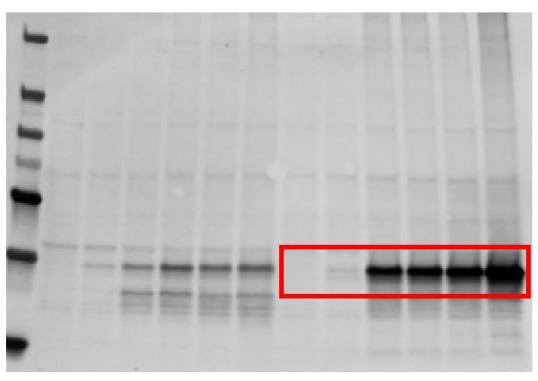

CELL:
anti-GAPDH (Proteintech Group, 60004-1-Ig)

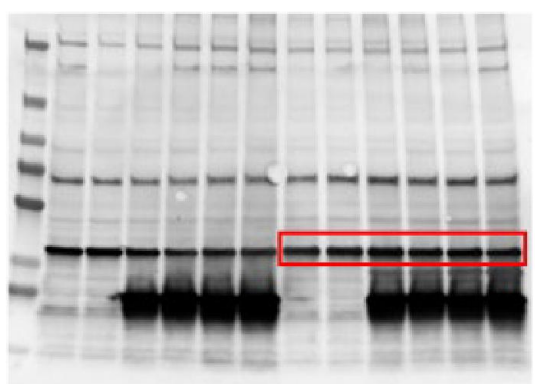

Supplementary Fig.6g

	Rep 1	Rep 2	Rep 3
Empty Vector	0.939414	1.280727	1.693701
WT	11.45635	11.00893	12.63222
D50K	9.911619	14.74149	15.26092
R52E	10.52286	9.337745	13.60982
W73A	14.66643	18.2237	15.39939

Supplementary Fig.9

Supplementary Fig.9c

CELL LYSATE:
anti-HA (Cell Signaling Technology, 3724)

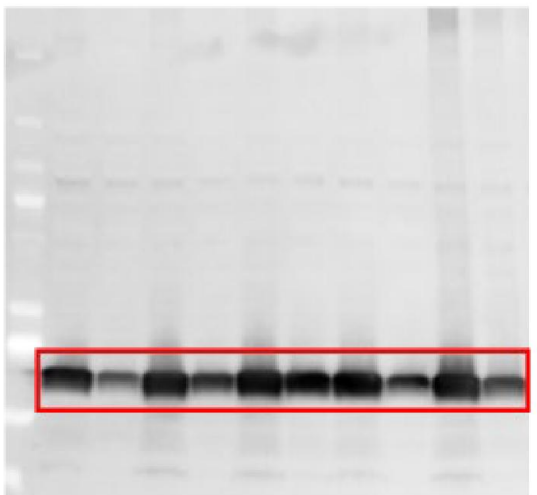

CELL LYSATE:
anti-FLAG (Sigma-Aldrich, F3165)

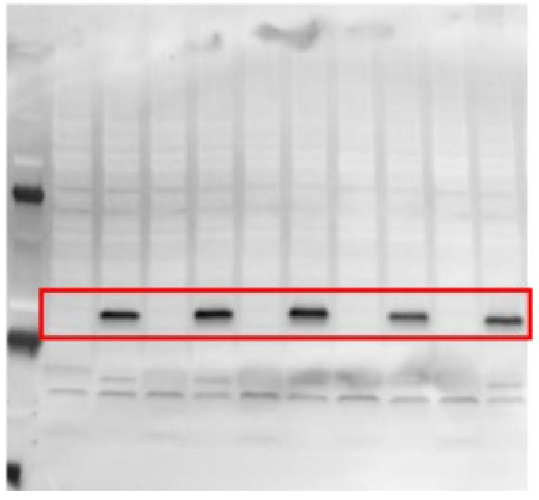

CELL LYSATE:
anti-GAPDH (Proteintech Group, 60004-1-Ig)

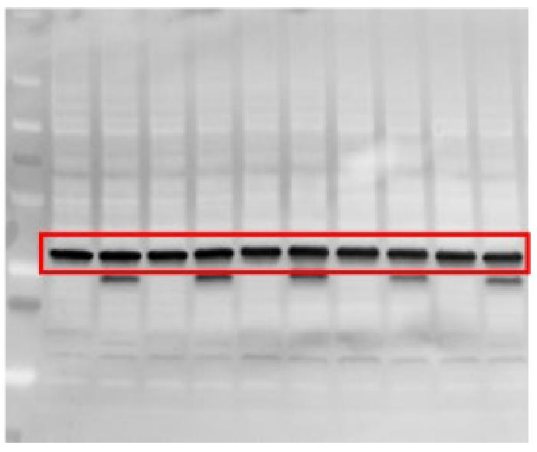

Supplement: Supplementary file 1 [file 41467_2026_75045_MOESM1_ESM.pdf]
