# Supplementary material for: The structural mechanism of HIV-2 Vif antagonism of human APOBEC3H
Source: Nat Commun. 2026 Jun 29;17:8079. doi: 10.1038/s41467-026-75045-1 (PMC13454587; doi:10.1038/s41467-026-75045-1)
Supplement: Supplementary file 2 [file 41467_2026_75045_MOESM2_ESM.pdf]

Description of Additional Supplementary Files

File name: Supplementary Movie 1
Description: HIV-2 Vif engages APOBEC3H through a binding mode distinct from that of HIV-1 Vif.
