# Supplementary material for: The structural mechanism of HIV-2 Vif antagonism of human APOBEC3H
Source: Nat Commun. 2026 Jun 29;17:8079. doi: 10.1038/s41467-026-75045-1 (PMC13454587; doi:10.1038/s41467-026-75045-1)
Supplement: Supplementary file 3 [file 41467_2026_75045_MOESM4_ESM.pdf]

nature portfolio

Corresponding author(s): John D. Gross

Last updated by author(s): Jun 10, 2026

Reporting Summary

Nature Portfolio wishes to improve the reproducibility of the work that we publish. This form provides structure for consistency and transparency in reporting. For further information on Nature Portfolio policies, see our Editorial Policies and the Editorial Policy Checklist.

Statistics

For all statistical analyses, confirm that the following items are present in the figure legend, table legend, main text, or Methods section.

n/a	Confirmed	
☐	☒	The exact sample size (*n*) for each experimental group/condition, given as a discrete number and unit of measurement
☐	☒	A statement on whether measurements were taken from distinct samples or whether the same sample was measured repeatedly
☐	☒	The statistical test(s) used AND whether they are one- or two-sided<br>*Only common tests should be described solely by name; describe more complex techniques in the Methods section.*
☒	☐	A description of all covariates tested
☒	☐	A description of any assumptions or corrections, such as tests of normality and adjustment for multiple comparisons
☐	☒	A full description of the statistical parameters including central tendency (e.g. means) or other basic estimates (e.g. regression coefficient) AND variation (e.g. standard deviation) or associated estimates of uncertainty (e.g. confidence intervals)
☐	☒	For null hypothesis testing, the test statistic (e.g. *F*, *t*, *r*) with confidence intervals, effect sizes, degrees of freedom and *P* value noted<br>*Give P values as exact values whenever suitable.*
☒	☐	For Bayesian analysis, information on the choice of priors and Markov chain Monte Carlo settings
☒	☐	For hierarchical and complex designs, identification of the appropriate level for tests and full reporting of outcomes
☒	☐	Estimates of effect sizes (e.g. Cohen's *d*, Pearson's *r*), indicating how they were calculated

Our web collection on statistics for biologists contains articles on many of the points above.

Software and code

Policy information about availability of computer code

Data collection	SerialEM-4.2
Data analysis	MotionCorr2 v1.3.0, CryoSPARC-v4.7.0, Pymol-v1.7.0, UCSF Chimera-1.15, UCSF Chimera X- 1.2.5, Coot-0.9.8, PHENIX-1.19.2, MEGA-X, Prism 6.0, AlphaFold 3, Adobe Illustrator 2020.

For manuscripts utilizing custom algorithms or software that are central to the research but not yet described in published literature, software must be made available to editors and reviewers. We strongly encourage code deposition in a community repository (e.g. GitHub). See the Nature Portfolio guidelines for submitting code & software for further information.

Data

Policy information about availability of data

All manuscripts must include a data availability statement. This statement should provide the following information, where applicable:

- Accession codes, unique identifiers, or web links for publicly available datasets
- A description of any restrictions on data availability
- For clinical datasets or third party data, please ensure that the statement adheres to our policy

The cryo-EM maps and atomic models of the HIV-2 Vif-A3H-CBFβ complex generated in this study have been deposited in the Electron Microscopy Data Bank (EMDB) and the Protein Data Bank (PDB). The monomeric complex is available under accession codes EMDB: EMD-74939 [https://www.ebi.ac.uk/pdbe/entry/emdb/EMD-74939] and PDB: 9ZY0 [http://doi.org/10.2210/pdb9ZY0/pdb], and the dimeric complex under EMDB: EMD-74940 [https://www.ebi.ac.uk/pdbe/entry/

emdb/EMD-74940] and PDB: 9ZY1[http://doi.org/10.2210/pdb9ZY1/pdb]. PDB entries (8FVI [http://doi.org/10.2210/pdb8FVI/pdb], 5W3V [http://doi.org/10.2210/pdb5W3V/pdb], 4N9F [http://doi.org/10.2210/pdb4N9F/pdb], 7ONI [http://doi.org/10.2210/pdb7ONI/pdb], 7B5M [http://doi.org/10.2210/pdb7B5M/pdb], 8CX0 [http://doi.org/10.2210/pdb8CX0/pdb], 6NIL [http://doi.org/10.2210/pdb6NIL/pdb]) used in this study were downloaded from Protein Data Bank.

Research involving human participants, their data, or biological material

Policy information about studies with human participants or human data. See also policy information about sex, gender (identity/presentation), and sexual orientation and race, ethnicity and racism.

Reporting on sex and gender	Not applicable
Reporting on race, ethnicity, or other socially relevant groupings	Not applicable
Population characteristics	Not applicable
Recruitment	Not applicable
Ethics oversight	Not applicable

Note that full information on the approval of the study protocol must also be provided in the manuscript.

Field-specific reporting

Please select the one below that is the best fit for your research. If you are not sure, read the appropriate sections before making your selection.

☒ Life sciences ☐ Behavioural & social sciences ☐ Ecological, evolutionary & environmental sciences

For a reference copy of the document with all sections, see nature.com/documents/nr-reporting-summary-flat.pdf

Life sciences study design

All studies must disclose on these points even when the disclosure is negative.

Sample size	The appropriate sample size was not predetermined in cryo-EM data analysis. The number of micrographs was increased until satisfactory map quality and resolution were achieved. The total number of micrographs collected was 9,809. The number of particles used for the final reconstruction was 1,257,210 for the dimeric structure and 545,960 for the monomeric structure, yielding well-resolved structural detail.
Data exclusions	Cryo-EM micrographs with ice or ethane contamination, empty carbon, and poor CTF fit (> 5 Å) were excluded manually. Particles belonging to bad classes were discarded and the data processing flowchart were summarized in Supplementary Figures. These criteria were pre-established and the procedure is a common practise in cryo-EM image analysis.
Replication	The A3H-VCBCC complex purification was repeated for at least 3 times and all attempts at replication were successful. The co-immunoprecipitation and A3H packaging assays were performed with two biological replicates, independent assays. Viral infectivity experiment was performed in biological triplicates (n=3) in order to allow for the calculation of mean values and the standard error of the mean.
Randomization	All cryo-EM particle images were randomly split into two half dataset for the estimation of overall resolution during Fourier Shell Correlation calculation. Otherwise, randomization was not relevant to this study.
Blinding	Blinding was not relevant to this study.

Reporting for specific materials, systems and methods

We require information from authors about some types of materials, experimental systems and methods used in many studies. Here, indicate whether each material, system or method listed is relevant to your study. If you are not sure if a list item applies to your research, read the appropriate section before selecting a response.

Materials & experimental systems

n/a	Involved in the study
☐	☒ Antibodies
☐	☒ Eukaryotic cell lines
☒	☐ Palaeontology and archaeology
☒	☐ Animals and other organisms
☒	☐ Clinical data
☒	☐ Dual use research of concern
☒	☐ Plants

Methods

n/a	Involved in the study
☒	☐ ChIP-seq
☒	☐ Flow cytometry
☒	☐ MRI-based neuroimaging

Antibodies

Antibodies used

Monoclonal Anti-FLAG M2 antibody produced in mouse, Sigma-Aldrich F3165 (1:5,000). Monoclonal Anti-HA antibody produced in rabbit, Cell Signaling Technology 3724 (1:5,000). Monoclonal Anti-Human Immunodeficiency Virus 1 (HIV-1) p24 antibody produced in mouse, BEI Resources ARP-3537 (1:5,000). Monoclonal Anti-human CBFβ antibody produced in mouse, Santa Cruz Biotechnology sc-166142 (1:1,000). Monoclonal Anti-GAPDH antibody produced in mouse, Proteintech Group, 60004-1-Ig (1:10,000). StarBright Blue 520 Goat Anti-Rabbit IgG, Bio-Rad 12005869 (1:5,000). StarBright Blue 700 Goat Anti-Mouse IgG, Bio-Rad 12004158 (1:5,000).

Validation

Antibody validations were performed by antibody suppliers.
Monoclonal Anti-FLAG M2 antibody: https://www.sigmaaldrich.com/US/en/product/sigma/f3165?srsltid=AfmBOopA-qnhWNIHlqq5pUgvT2nxEiR_iOM2BcSkA_tgDEqUpJLA6-9Z
Monoclonal Anti-HA antibody: https://www.cellsignal.com/products/primary-antibodies/ha-tag-c29f4-rabbit-monoclonal-antibody/3724?srsltid=AfmBOorD-CfEWi4uDho_vVBpZdruHEygz-n08n35bkcg73ru5SjgTp6W
Monoclonal Anti-Human Immunodeficiency Virus 1 (HIV-1) p24 antibody: https://www.beiresources.org/Catalog/BEIMonoclonalAntibodies/ARP-3537.aspx
Monoclonal Anti-human CBFβ antibody: https://www.scbt.com/p/pebp2beta-antibody-a-4
Monoclonal Anti-GAPDH antibody: https://www.ptglab.com/products/GAPDH-Antibody-60004-1-Ig.htm?srsltid=AfmBOorCO_FKpLxOLb8U4MT-SGItjZmSyWXL-gvmt4e4XKWRQKS3DWDO
StarBright Blue 520 Goat Anti-Rabbit IgG: https://www.bio-rad.com/en-us/sku/12005869-starbright-blue-520-goat-anti-rabbit-igg-400-ul?ID=12005869
StarBright Blue 700 Goat Anti-Mouse IgG: https://www.bio-rad.com/en-us/sku/12004158-starbright-blue-700-goat-anti-mouse-igg-400-ul?ID=12004158

Eukaryotic cell lines

Policy information about cell lines and Sex and Gender in Research

Cell line source(s)

Sf9 insect cells were obtained from Thermo Fisher Scientific (Gibco, Cat. No. 12659017). HEK293T and HEK293T/17 cell lines were obtained from ATCC (Cat. Nos. CRL-3216 and CRL-11268, respectively). The SupT1 cell line was obtained from the NIH AIDS Reagent Program (ARP-100).

Authentication

None of the cell line used was authenticated.

Mycoplasma contamination

All cell lines were tested negative for mycoplasma contamination by the authors of this study.

Commonly misidentified lines
(See ICLAC register)

No commonly misidentified lines were used in this study.

Plants

Seed stocks

Not applicable

Novel plant genotypes

Not applicable

Authentication

Not applicable
