# Supplementary material for: The structural mechanism of HIV-2 Vif antagonism of human APOBEC3H
Source: Nat Commun. 2026 Jun 29;17:8079. doi: 10.1038/s41467-026-75045-1 (PMC13454587; doi:10.1038/s41467-026-75045-1)
Supplement: Supplementary file 4 [file 41467_2026_75045_MOESM5_ESM.pdf]

nature portfolio

Peer Review File

The structural mechanism of HIV-2 Vif antagonism of human APOBEC3H

Corresponding Author: Professor John Gross

This file contains all reviewer reports in order by version, followed by all author rebuttals in order by version.

Attachments originally included by the reviewers as part of their assessment can be found at the end of this file.

Version 0:

Reviewer comments:

Reviewer #1

(Remarks to the Author)
See attached file

Reviewer #2

(Remarks to the Author)
The manuscript by Niu et al describes the cryo-EM structure of human APOBEC3H in complex with HIV-2 Vif and interaction partners – VCBC with Rbx2-Cullin5. The authors have a better resolution of the totality of the complex for HIV-2 Vif in complex with APOBEC3H than the two previous papers detailing structures of HIV-1 Vif in complex with APOBEC3H (PMID: 40593686, 37640699). The structure reveals that the dimer of APOBEC3H remains intact and each interacts with VCBC. Structure-function studies show that the proximal APOBEC3H gets ubiquitinated. It has been suggested in the literature that HIV-2 Vif is more potent at inducing degradation than HIV-1 Vif.

The most noteworthy result is the identification of the HIV-1-Vif interface that interacts with APOBEC3H. The cryo-EM structure confirms the key residues originally identified in PMID: 24942576 (2014), but provide the full detail that was not possible with mutagenesis. The data in the manuscript provides a rationale for increased activity against APOBEC3H due to the larger Vif APOBEC3H interface of HIV-2 Vif with APOBEC3H than HIV-1 Vif. However, it is not known if this rationale applies to other APOBEC3s or it is only applicable to APOBEC3H.

Minor corrections:
1. Line 29: use of extensive G-to-A hypermutation in sentence. The extensive G-to-A hypermutation only occurs after multiple rounds of infection in the presence of Vif. There is only hypermutation in the absence of Vif. PMID: 31025011.

2. Line 312: regarding mention of caveat. May want to clarify that the cryo-EM structures used NL4-3 Vif that is not optimized for APOBEC3H degradation.

Scientific comments:
1. Figure 3 and CBFB mutant W73A. The difference between W73A and R52E mutants may be that the with the W73A mutant Vif is destabilized in the cell lysate, but not with the R52E mutant. Nonetheless, the destabilized Vif still interacts with APOBEC3H. This interaction may be blocking the encapsidation. More fulsome discussion of the immunoblot data and testing this possibility would be useful to understanding the interaction.

2. Line 326-327: “This observation suggests that A3H degradation and packaging are mechanistically distinct processes.” I think this statement should be removed or clarified. I think this is known. Do the authors mean that when A3H is not degraded that Vif may inhibit packaging by a different mechanism? This review summarizes evidence for competition of RNA binding as a mechanism by which Vif can inhibit APOBEC3G encapsidation independent of degradation (PMID: 33916704). The authors should investigate this as it may provide more insight into the APOBEC3H and Vif dynamics in the absence of CBF-B.

Reviewer #3

(Remarks to the Author)
Overview

This study by Niu et al. seeks to evaluate the structural basis of HIV-2 Vif-mediated antagonism of human A3H proteins. Using a combination of cryo-EM structural analyses and biochemical/virologic assays, the authors propose a mechanistic molecular model for HIV-2 Vif-mediated antagonism of A3H. The manuscript is well written, and the work provides valuable insights to the field.

While the structural analyses are central to the study, my review focused primarily on the molecular virology and functional experiments. Overall, these experiments were methodologically sound and appropriately controlled, and the data broadly support the authors' conclusions. My comments below are primarily aimed at clarifying the mechanistic context and improving consistency and presentation.

Major comments

The authors leverage structural data to generate Vif mutants that disrupt A3H interaction and antiviral activity, and they reference a double-stranded RNA (dsRNA) binding mechanism previously reported in the literature. However, the manuscript would benefit from clearer integration of these findings with the known mechanistic functions of A3H.

Specifically, the authors should expand the discussion to address:
1.the established mechanisms of A3H antiviral activity (e.g., inhibition of reverse transcription and/or cytidine deamination), and
2.the functional role of dsRNA binding in A3H activity and how this may relate to the Vif interaction described here.

Providing this context would help place the structural and virologic findings into a more complete mechanistic framework.

Minor comments

Methods used to quantify reverse transcriptase activity for virus normalization are not described. Please include sufficient detail to allow reproducibility.

Methods: Vendor information for all antibodies should be provided, as the reported dilutions are likely vendor specific.

Lines 147–151: The authors state that the "Trp-rich motif is conserved in SIVsmm Vif." Please provide supporting evidence (e.g., sequence alignment or citation).

Several inconsistencies were noted across figures and should be reviewed for clarity and consistency:
• "hA3H" nomenclature is used throughout, but differs in Fig. 4a, 4b, 4k (and possibly others).
• Fig. 4j: "Cell" should match labeling in other panels.
• Inconsistent terminology for controls: "No Vif," "Empty Vector," and "dVif" (e.g., Figs. 2f, 3f, Extended Data Fig. 1).

Fig. 4 legend: Please specify which HIV-1 Vif construct was used.

The authors suggest that targeting Vif–A3 interactions may represent a therapeutic strategy. Given that this study focuses on HIV-2, it would be helpful to either (1) provide brief context regarding the global burden and clinical relevance of HIV-2 infection, or (2) temper the therapeutic framing to better align with the scope of the current work.

Version 1:

Reviewer comments:

Reviewer #1

(Remarks to the Author)
The authors have thoughtfully responded to my comments on their original manuscript.

Reviewer #2

(Remarks to the Author)
The authors have answered all of my previous comments.

Reviewer #3

(Remarks to the Author)
The authors have adequately addressed this reviewer's comments from the first-round submission.

Open Access This Peer Review File is licensed under a Creative Commons Attribution 4.0 International License, which permits use, sharing, adaptation, distribution and reproduction in any medium or format, as long as you give appropriate credit to the original author(s) and the source, provide a link to the Creative Commons license, and indicate if changes were made.
In cases where reviewers are anonymous, credit should be given to 'Anonymous Referee' and the source.
The images or other third party material in this Peer Review File are included in the article's Creative Commons license, unless indicated otherwise in a credit line to the material. If material is not included in the article's Creative Commons license and your intended use is not permitted by statutory regulation or exceeds the permitted use, you will need to obtain permission directly from the copyright holder.
To view a copy of this license, visit https://creativecommons.org/licenses/by/4.0/

We greatly appreciate the time, effort and constructive comments of reviewers. Please see our point-by-point responses below (colored in dark blue).

Reviewer #1 (Remarks to the Author)

APOBEC3 (A3) proteins are cytidine deaminases. Some of them (A3F, A3G, A3H) restrict retroviruses by binding RNA in virions and hypermutating the single-stranded DNA product of viral reverse transcriptase. To block this restriction, the viral protein Vif recognizes host A3 proteins and recruits a Cullin-RING E3 ligase for hyper-ubiquitination and proteosomal destruction of the A3. The paper describes a 3.2-A cryo-EM structure of the human A3H with a complex of HIV-2 Vif and human CBFβ, Elongin B, Elongin C and Cullin-5 N-terminal domain.

Vif proteins are specific for A3s of the hosts infected by the producing virus; the mechanism of Vif-A3 recognition has been enigmatic. Two earlier studies by other labs revealed the basis of HIV-1 Vif recognition of A3H. Here, the most important finding is that HIV-2 Vif recognizes a distinct site on human A3H that does not overlap the site for HIV-1 Vif recognition. The work is of high quality, and the interpretation of the EM maps is on solid ground.

The full E3 ligase complex was modeled onto the structure to identify Lys side chains where A3H could be ubiquitinated (27, 50, 51, 52, 64, 153). Among these, all but Lys64 are known A3H ubiquitination sites. Thus, a second potentially significant finding is the possibility that HIV-2 Vif leads to A3H Lys64 ubiquitination. The paper would be strengthened by testing this possibility.

Response: Thank you for your insightful comments. We have assessed the potential ubiquitination sites by introducing lysine to arginine mutations on A3H, and the degradation profile of A3H mutants is now included in the revised Supplementary Fig.9c. Mutations within the K-rich motif largely impaired HIV-2 Vif-mediated A3H degradation, indicating that this region serves as the primary ubiquitination site for HIV-2 Vif and represents a conserved ubiquitination site required for both HIV-1 and HIV-2 Vif-mediated A3H degradation. The K27R and K64R mutants exhibited partial resistance to HIV-2 Vif-mediated degradation, suggesting that K27 and K64 also contribute as additional ubiquitination sites. We have included these discussions from line 246 to line 252 in the revised manuscript.

Strengths of the current study are the use of full-length wild-type hA3H and HIV-2 Vif and the absence of chemical crosslinking. Have the authors considered whether/how the reported structures of hA3H with HIV-1 Vif may be influenced by the crosslinking used in previous studies? Additionally, a K97Q substitution in hA3H was used in one of the structures with HIV-1 Vif. Gln97 is buried in the HIV-1 Vif interface near Vif residues His56, Lys63, Arg90 and Lys92 – a very unlikely position for the wild type hA3H Lys97.

Response: We appreciate the reviewer noting that our studies on HIV-2 Vif and A3H were carried out with full-length and wild-type proteins. The reported HIV-1 Vif and A3H structures required engineering - cross-linking and mutagenesis or using cross-

linking of HIV-1 Vif to non-cognate A3H from chimpanzee. Both of these structures contain a similar Vif-A3H interface. The simplest interpretation is that this interaction reflects the HIV-1 Vif structure bound to chimpanzee A3H. However, the molecular details of how HIV-1 Vif engages wild-type human A3H remain to be fully elucidated. Importantly, there are biological differences between human A3H and chimpanzee A3H, for example, in intracellular localization and responses to primate lentiviral Vif (PMID: 21653666; PMID: 29267382).

In detail, human A3H contains a lysine at position 97, whereas chimpanzee A3H has a glutamine at this position. A K97Q substitution was required to form the HIV-1 Vif-hA3H complex in a reported structure (PMID: 37640699), suggesting that Q97 plays a critical role in mediating the interaction. Consistently, that structure shows Q97 interacting with K63 of HIV-1 Vif, indicating that the complex more closely resembles the HIV-1 Vif-chimpanzee A3H interaction. This is further supported by another structure of HIV-1 Vif bound to chimpanzee A3H (PMID: 40593686), which shows a similar interaction mode. Therefore, as the reviewer implies it is possible that the published structures of HIV-1 Vif to modified A3H should be tempered in light our HIV-2 Vif to full-length unmodified hA3H. We have revised the discussion in the revised manuscript from lines 310-315 to further address these caveats.

p.14: This Discussion sentence is unclear to me: “Unexpectedly, the CBFβ W73A mutation impairs HIV-2 Vif induced degradation of A3H, without noticeably restoring A3H packaging.” Does “Vif induced degradation” refer to the last column/lane in Fig 3d? The co-IP results were weak for this experiment (+Vif +CBFβ/W73A +A3H), so it’s difficult to draw any conclusion. In the presented structure, CBFβ Trp73 interacts with both hA3H and HIV-2 Vif, where it is part of an aromatic cluster with Trp49 and Trp51 in the Vif WAWW motif. Some of the effects of CBFβ/W73A may be due to a weakened interaction with Vif. Vif is notoriously unstable without CBFβ, so reduced Vif levels due to reduced CBFβ affinity should be a consideration here. Levels of HIV-2 Vif in cells are very low in absence of CBFβ (Fig 3e 2nd column, +Vif -CBFβ), and reduced in CBFβ/W73A (Fig 3d last column – reduced Vif in the IP and in the input). While the main-chain trace of both Vif and CBFβ and the positions of the indole side chains are clear in the EM map, these amino acids are on loops where the structural changes due mutagenesis are difficult or impossible to predict, i.e. how CBFβ/W73A or HIV-2 Vif/WAW49-51ASA affect Vif-CBFβ affinity.

Response: Thank you for your critical comments on this. To clarify, “Vif induced degradation” refers to the last column/lane in Fig.3e rather than Fig.3d. We agree that the CBFβ W73A may reduce Vif stability as shown in Fig.3d. The effects of CBFβ W73A on A3H degradation and packaging in Fig.3e could result from reduced interaction with Vif or from degradation-independent mechanisms. Previous studies on HIV-1 Vif and A3G has demonstrated that Vif inhibits A3G encapsidation by competing with RNA binding or affecting its multimerization (reviewed in PMID: 33916704). The reduced A3H degradation without increasing A3H packaging by CBFβ W73A in Fig.3e suggests that Vif may inhibit A3H encapsidation through degradation-independent mechanisms. We have revised the discussion in the revised manuscript from line 329 to line 335.

Other points

Please state clearly that the sequences of all protein components of the deposited structures are wild type.

Response: We have clarified in both the Results and Methods sections of the revised manuscript that all components in the deposited structures are wild type.

The negatively charged pocket in hA3H that's occupied by CBFβ Arg52 in the HIV-2 Vif complex is a nice observation. HIV-1 Vif Lys92 binds in the same A3H pocket – could the be a general feature of A3H-Vif interaction?

Response: Thank you for pointing this out. It is possible that this pocket represents a hotspot for protein-protein interface in Vif-A3H complex. We have included the discussion in the revised manuscript from line 324 to line 326.

The hA3H Asn103 interaction with Vif Trp51 is more extensive than any interaction of Asn103 with CBFβ Trp73.

Response: Yes, that is correct. hA3H N103 is sandwiched between W51 of Vif and W73 of CBFβ, with a shorter distance to W51 than to W73. Accordingly, the interaction between N103 and W51 is likely stronger than that between N103 and W73.

I find a few missed or mis-identified inter-protein contacts.

CBFβ Arg52 NH1 to C=O of A3H His99 (3.0 A)

CBFβ Arg52 NH2 to C=O of A3H Ile96 (3.3 A)

but probably not to OE of Gln130 (3.7 A)

Response: Thank you for the careful examination of our structure. We have incorporated these interactions into the revised Fig. 3b-c and described them in the revised manuscript from line 152 to line 153.

p.20-21: Methods for co-IP of A3H or CBFβ with Vif and for immunoblot of A3H incorporation into VLPs, explain the data in Fig 2d-e, but not Fig 3d-e & Fig S6e-f. There is no mention of the CBFβ-Myc plasmid, and presumably HEK293T-shCBFβ cells were used for the data in Fig 3d-e and Fig S6e-f.

Response: We have revised the Methods section and added CBFβ-Myc plasmid information in the revised manuscript, and clarified the use of HEK293T-shCBFβ in these experiments.

In the co-IP assays (Figs 2d, 3d, 4i, S5a, S6e), it appears that the "input" blots were done before combining the lysates of HEK293T cells (Vif & CBFβ) and HEK293T-shCBFβ cells (A3H). Please state clearly.

Response: We have clarified in the revised manuscript that input samples were collected prior to incubation with the beads.

p.15 unclear: "The A3H CDA domain occupies the space between the two CDA domains of A3G, thereby establishing a distinct binding mode (Extended Data Fig.10e)."

Surely you mean "In the complex with Vif-CBFβ, the A3H CDA domain occupies the space…"

Response: We have revised this sentence in the revised manuscript to improve clarity.

Reviewer #2 (Remarks to the Author)

The manuscript by Niu et al describes the cryo-EM structure of human APOBEC3H in complex with HIV-2 Vif and interaction partners – VCBC with Rbx2-Cullin5. The authors have a better resolution of the totality of the complex for HIV-2 Vif in complex with APOBEC3H than the two previous papers detailing structures of HIV-1 Vif in complex with APOBEC3H (PMID: 40593686, 37640699). The structure reveals that the dimer of APOBEC3H remains intact and each interacts with VCBC. Structure-function studies show that the proximal APOBEC3H gets ubiquitinated. It has been suggested in the literature that HIV-2 Vif is more potent at inducing degradation than HIV-1 Vif.

The most noteworthy result is the identification of the HIV-1-Vif interface that interacts with APOBEC3H. The cryo-EM structure confirms the key residues originally identified in PMID: 24942576 (2014), but provide the full detail that was not possible with mutagenesis. The data in the manuscript provides a rationale for increased activity against APOBEC3H due to the larger Vif APOBEC3H interface of HIV-2 Vif with APOBEC3H than HIV-1 Vif. However, it is not known if this rationale applies to other APOBEC3s or it is only applicable to APOBEC3H.

Response: Thank you for your critical comments. The structure of the HIV-2 Vif-hA3H complex presented in this study suggests that an expanded interaction interface between HIV-2 Vif and hA3H may contribute to enhanced Vif-mediated antagonism of hA3H. However, this is the first reported structure of an HIV-2 Vif in complex with any APOBEC3 protein. Therefore, in the absence of structural information for HIV-2 Vif bound to other APOBEC3 family members, we cannot conclude that the breadth of HIV-2 Vif antagonism across APOBEC3 proteins is generally driven by an enlarged interaction interface. Addressing this question will require additional structures of HIV-2 Vif in complex with other APOBEC3 proteins.

Minor corrections:

1. Line 29: use of extensive G-to-A hypermutation in sentence. The extensive G-to-A hypermutation only occurs after multiple rounds of infection in the presence of Vif. There is only hypermutation in the absence of Vif. PMID: 31025011.

Response: We have revised the wording accordingly in the revised manuscript.

2. Line 312: regarding mention of caveat. May want to clarify that the cryo-EM structures

used NL4-3 Vif that is not optimized for APOBEC3H degradation.

Response: Yes, this is correct and Reviewer #1 had a similar comment. We have expanded this caveat in the revised manuscript, as previous studies did not use an optimized Vif or authentic full-length, unmodified human A3H.

Scientific comments:

1. Figure 3 and CBFB mutant W73A. The difference between W73A and R52E mutants may be that the with the W73A mutant Vif is destabilized in the cell lysate, but not with the R52E mutant. Nonetheless, the destabilized Vif still interacts with APOBEC3H. This interaction may be blocking the encapsidation. More fulsome discussion of the immunoblot data and testing this possibility would be useful to understanding the interaction.

Response: We agree that the CBFβ W73A may reduce Vif stability. CBFβ W73 forms an aromatic cluster with W49 and W51 of HIV-2 Vif. The increased steady-state level of A3H may be due to reduced HIV-2 Vif stability caused by the CBFβ W73A mutation, which weakens its interaction with Vif. Consistently, we noticed the reduced Vif in the INPUT and IP in Fig.3d. The elevated A3H is not incorporated into VLP, suggesting a degradation-independent mechanism by which HIV-2 Vif inhibits A3H encapsidation. We have included this discussion in the revised manuscript from line 329 to line 335.

2. Line 326-327: "This observation suggests that A3H degradation and packaging are mechanistically distinct processes." I think this statement should be removed or clarified. I think this is known. Do the authors mean that when A3H is not degraded that Vif may inhibit packaging by a different mechanism? This review summarizes evidence for competition of RNA binding as a mechanism by which Vif can inhibit APOBEC3G encapsidation independent of degradation (PMID: 33916704). The authors should investigate this as it may provide more insight into the APOBEC3H and Vif dynamics in the absence of CBF-B.

Response: Thank you for your insightful comments. We clarify that because the CBFβ W73A mutation impairs HIV-2 Vif induced degradation of A3H, without noticeably restoring A3H packaging (Fig 3e), we conclude that Vif also inhibits A3H restriction through a degradation-independent mechanism. Accordingly, we have revised the Discussion to state: "The elevated A3H is not incorporated into VLPs, suggesting a degradation-independent mechanism by which HIV-2 Vif inhibits A3H encapsidation." We have cited the recent review paper to point the reader to degradation-independent mechanisms of Vif antagonism of A3G. However, we feel that the analysis of A3H and Vif dynamics in the absence of CBFβ is beyond the scope of the present study and will be addressed in future work.

Reviewer #3 (Remarks to the Author):

Overview

This study by Niu et al. seeks to evaluate the structural basis of HIV-2 Vif-mediated antagonism of human A3H proteins. Using a combination of cryo-EM structural analyses and biochemical/virologic assays, the authors propose a mechanistic molecular model for

HIV-2 Vif-mediated antagonism of A3H. The manuscript is well written, and the work provides valuable insights to the field.

While the structural analyses are central to the study, my review focused primarily on the molecular virology and functional experiments. Overall, these experiments were methodologically sound and appropriately controlled, and the data broadly support the authors' conclusions. My comments below are primarily aimed at clarifying the mechanistic context and improving consistency and presentation.

Major comments

The authors leverage structural data to generate Vif mutants that disrupt A3H interaction and antiviral activity, and they reference a double-stranded RNA (dsRNA) binding mechanism previously reported in the literature. However, the manuscript would benefit from clearer integration of these findings with the known mechanistic functions of A3H.

Specifically, the authors should expand the discussion to address:

1.the established mechanisms of A3H antiviral activity (e.g., inhibition of reverse transcription and/or cytidine deamination), and

2.the functional role of dsRNA binding in A3H activity and how this may relate to the Vif interaction described here.

Providing this context would help place the structural and virologic findings into a more complete mechanistic framework.

Response: Thank you for your insightful comments. In addition to its cytosine deaminase activity, A3H restricts viral replication through deaminase-independent mechanisms, including incorporation into viral particles and inhibition of reverse transcription (PMID: 31578294, PMID: 29290613). RNA binding promotes A3H encapsidation and suppresses reverse transcription, suggesting that RNA-mediated A3H dimer formation represents a particularly potent antiviral state (PMID: 29290613). HIV-2 Vif counteracts A3H more efficiently by targeting each A3H protomer for proteasomal degradation. This mechanism may partially account for the stronger antagonistic activity of HIV-2 Vif against A3H compared to HIV-1 Vif, which mediates ubiquitination and degradation of only the proximal A3H protomer (PMID: 40593686). We have cited and discussed these papers in the revised manuscript from line 283 to line 290.

Minor comments

Methods used to quantify reverse transcriptase activity for virus normalization are not described. Please include sufficient detail to allow reproducibility.

Response: We have provided a detailed description of the reverse transcriptase activity normalization in the Methods section from line 509 to line 518.

Methods: Vendor information for all antibodies should be provided, as the reported dilutions are likely vendor specific.

Response: The vendor and catalog information of antibodies have been added in the revised manuscript.

Lines 147–151: The authors state that the “Trp-rich motif is conserved in SIVsmm Vif.” Please provide supporting evidence (e.g., sequence alignment or citation).

Response: We have included a WebLogo plot in the revised Supplementary Fig. 5a to illustrate the conservation of the Trp-rich motif in SIVsmm Vif.

Several inconsistencies were noted across figures and should be reviewed for clarity and consistency:

• "hA3H" nomenclature is used throughout, but differs in Fig. 4a, 4b, 4k (and possibly others).

• Fig. 4j: "Cell" should match labeling in other panels.

• Inconsistent terminology for controls: "No Vif," "Empty Vector," and "dVif" (e.g., Figs. 2f, 3f, Extended Data Fig. 1).

Response: We have clarified and ensured that labels are consistent across figures.

Fig. 4 legend: Please specify which HIV-1 Vif construct was used.

Response: We have indicated that HIV-1 Vif used in Fig.4 was from LAI strain.

The authors suggest that targeting Vif–A3 interactions may represent a therapeutic strategy. Given that this study focuses on HIV-2, it would be helpful to either (1) provide brief context regarding the global burden and clinical relevance of HIV-2 infection, or (2) temper the therapeutic framing to better align with the scope of the current work.

Response: Approximately 2 million people worldwide are infected with HIV-2, which is intrinsically resistant to non-nucleoside reverse transcriptase inhibitors and shows reduced susceptibility to some protease inhibitors used for HIV-1 treatment (PMID: 34832533). Therapeutic options for HIV-2 are therefore more limited than for HIV-1. Consistent with this, lenacapavir, the first approved capsid inhibitor for HIV-1, exhibits over 10-fold lower potency against HIV-2, with most patients developing capsid-associated drug resistance mutations (PMID: 38060982; PMID: 39737782). This study provides a rationale for developing antiviral therapeutics that target Vif-A3 interactions. We have incorporated this discussion into the revised manuscript from line 357 to line 363.

APOBEC3 (A3) proteins are cytidine deaminases. Some of them (A3F, A3G, A3H) restrict retroviruses by binding RNA in virions and hypermutating the single-stranded DNA product of viral reverse transcriptase. To block this restriction, the viral protein Vif recognizes host A3 proteins and recruits a Cullin-RING E3 ligase for hyper-ubiquitination and proteosomal destruction of the A3. The paper describes a 3.2-Å cryo-EM structure of the human A3H with a complex of HIV-2 Vif and human CBFβ, Elongin B, Elongin C and Cullin-5 N-terminal domain.

Vif proteins are specific for A3s of the hosts infected by the producing virus; the mechanism of Vif-A3 recognition has been enigmatic. Two earlier studies by other labs revealed the basis of HIV-1 Vif recognition of A3H. Here, the most important finding is that HIV-2 Vif recognizes a distinct site on human A3H that does not overlap the site for HIV-1 Vif recognition. The work is of high quality, and the interpretation of the EM maps is on solid ground.

The full E3 ligase complex was modeled onto the structure to identify Lys side chains where A3H could be ubiquitinated (27, 50, 51, 52, 64, 153). Among these, all but Lys64 are known A3H ubiquitination sites. Thus, a second potentially significant finding is the possibility that HIV-2 Vif leads to A3H Lys64 ubiquitination. The paper would be strengthened by testing this possibility.

Strengths of the current study are the use of full-length wild-type hA3H and HIV-2 Vif and the absence of chemical crosslinking. Have the authors considered whether/how the reported structures of hA3H with HIV-1 Vif may be influenced by the crosslinking used in previous studies? Additionally, a K97Q substitution in hA3H was used in one of the structures with HIV-1 Vif. Gln97 is buried in the HIV-1 Vif interface near Vif residues His56, Lys63, Arg90 and Lys92 – a very unlikely position for the wild type hA3H Lys97.

p.14: This Discussion sentence is unclear to me: “Unexpectedly, the CBFβ W73A mutation impairs HIV-2 Vif induced degradation of A3H, without noticeably restoring A3H packaging.” Does “Vif induced degradation” refer to the last column/lane in Fig 3d? The co-IP results were weak for this experiment (+Vif +CBFβ/W73A +A3H), so it’s difficult to draw any conclusion. In the presented structure, CBFβ Trp73 interacts with both hA3H and HIV-2 Vif, where it is part of an aromatic cluster with Trp49 and Trp51 in the Vif WAWW motif. Some of the effects of CBFβ/W73A may be due to a weakened interaction with Vif. Vif is notoriously unstable without CBFβ, so reduced Vif levels due to reduced CBFβ affinity should be a consideration here. Levels of HIV-2 Vif in cells are very low in absence of CBFβ (Fig 3e 2nd column, +Vif -CBFβ), and reduced in CBFβ/W73A (Fig 3d last column – reduced Vif in the IP and in the input). While the main-chain trace of both Vif and CBFβ and the positions of the indole side chains are clear in the EM map, these amino acids are on loops where the structural changes due mutagenesis are difficult or impossible to predict, i.e. how CBFβ/W73A or HIV-2 Vif/WAW49-51ASA affect Vif-CBFβ affinity.

Other points

Please state clearly that the sequences of all protein components of the deposited structures are wild type.

The negatively charged pocket in hA3H that’s occupied by CBFβ Arg52 in the HIV-2 Vif complex is a nice observation. HIV-1 Vif Lys92 binds in the same A3H pocket – could the be a general feature of A3H-Vif interaction?

The hA3H Asn103 interaction with Vif Trp51 is more extensive than any interaction of Asn103 with CBFβ Trp73.

I find a few missed or mis-identified inter-protein contacts.

CBFβ Arg52 NH1 to C=O of A3H His99 (3.0 Å)
CBFβ Arg52 NH2 to C=O of A3H Ile96 (3.3 Å)
but probably not to OE of Gln130 (3.7 Å)

p.20-21: Methods for co-IP of A3H or CBFβ with Vif and for immunoblot of A3H incorporation into VLPs, explain the data in Fig 2d-e, but not Fig 3d-e & Fig S6e-f. There is no mention of the CBFβ-Myc plasmid, and presumably HEK293T-shCBFβ cells were used for the data in Fig 3d-e and Fig S6e-f.

In the co-IP assays (Figs 2d, 3d, 4i, S5a, S6e), it appears that the "input" blots were done before combining the lysates of HEK293T cells (Vif & CBFβ) and HEK293T-shCBFβ cells (A3H). Please state clearly.

p.15 unclear: "The A3H CDA domain occupies the space between the two CDA domains of A3G, thereby establishing a distinct binding mode (Extended Data Fig.10e)."
Surely you mean "In the complex with Vif-CBFβ, the A3H CDA domain occupies the space…"
